# Supplementary material for: Three rate-determining protein roles in photosynthetic O2-evolution addressed by time-resolved experiments on genetically modified photosystems
Source: Nat Commun. 2025 Oct 28;16:9515. doi: 10.1038/s41467-025-64513-9 (PMC12569156; doi:10.1038/s41467-025-64513-9)
Supplement: Supplementary file 1 — Supplementary Information [file 41467_2025_64513_MOESM1_ESM.pdf]

Supplementary Information

Three rate-determining protein roles in photosynthetic O_2-evolution addressed by time-resolved experiments on genetically modified photosystems

Sarah M. Mäusle[1], Gianluca Parisse[2], Ricardo Assunção[1], Cristina De Santis[2], Philipp S. Simon[1,3], Daniele Narzi[2], Leonardo Guidoni[2], Richard J. Debus[4], and Holger Dau[1]

1. Department of Physics, Freie Universität Berlin, Germany
2. Department of Physical and Chemical Sciences, University of L'Aquila, Italy
3. Molecular Biophysics and Integrated Bioimaging Division, Lawrence Berkeley National, Laboratory, USA
4. Department of Biochemistry, University of California, Riverside, USA

Table of Contents

1. Verification of site mutations by mass spectrometry 2

2. Additional information on the O_2 polarography data, Supplementary Equations 5

3. Static FTIR difference spectra 7

4. Time-resolved IR: exp. details, fit results, additional data, Suppl. Discussion 9

5. Molecular Dynamics: methods, additional analyses 23

6. References.. 32

1. Verification of site mutations by mass spectrometry.

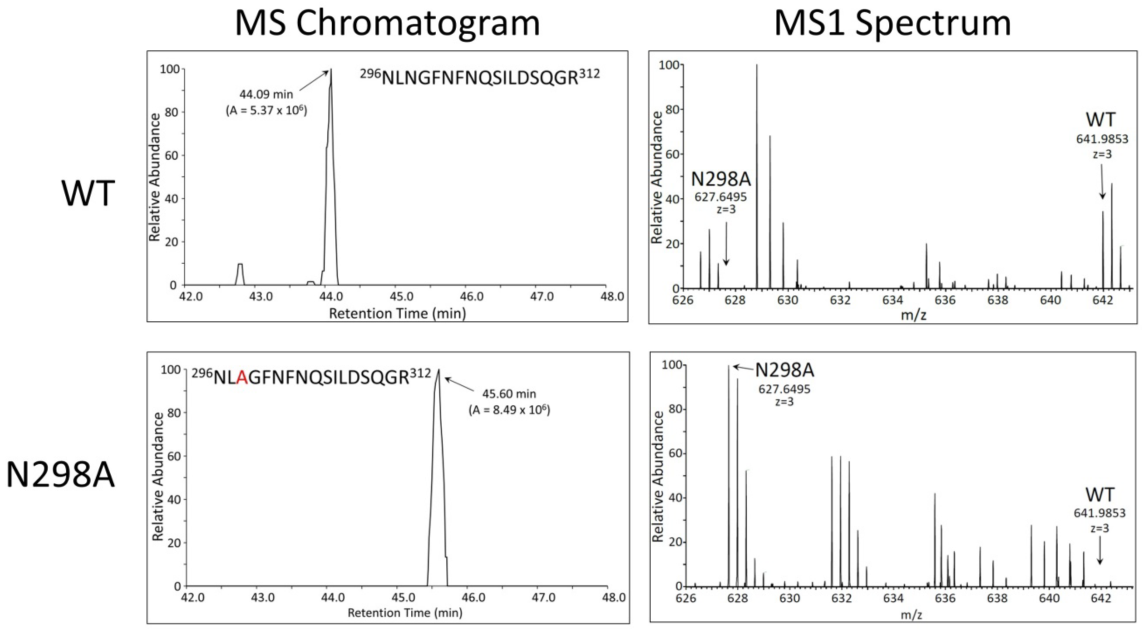

Supplementary Fig. 1 Comparison of the MS chromatograms and MS1 spectra of the chymotryptic/tryptic peptide purified from WT and D1-N298A PSII core complexes that contains position 298 of the D1 subunit. At least 99% of the peptide from D1-N298A contained the desired Ala residue (red). The peaks near m/z 632 and 636-641 are from unidentified peptides that do not include position 298 of the D1 subunit. In the MS1 spectra, the difference between the WT peak at 641.9853 (z = 3) and the N298A peak at 627.6495 (z = 3) shows a mass decrease of 43 Da in the mutant as expected from changing Asn to Ala at position 298.

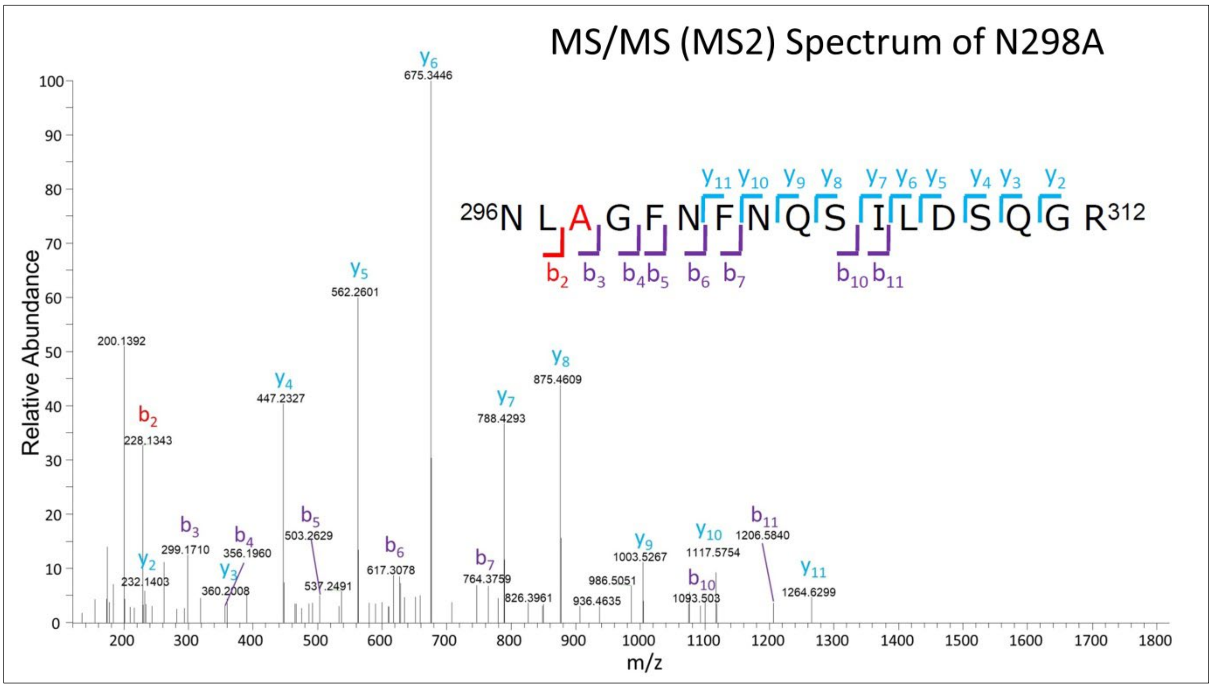

Supplementary Fig. 2 The MS/MS spectrum of the chymotryptic/tryptic peptide purified from D1-N298A PSII core complexes that contains position 298 of the D1 subunit. The masses of the *b* fragments *b*3, *b*4, *b*5, *b*6, *b*7, *b*10, and *b*11 (violet) confirm the identity of the peptide and the presence of the D1-N298A mutation.

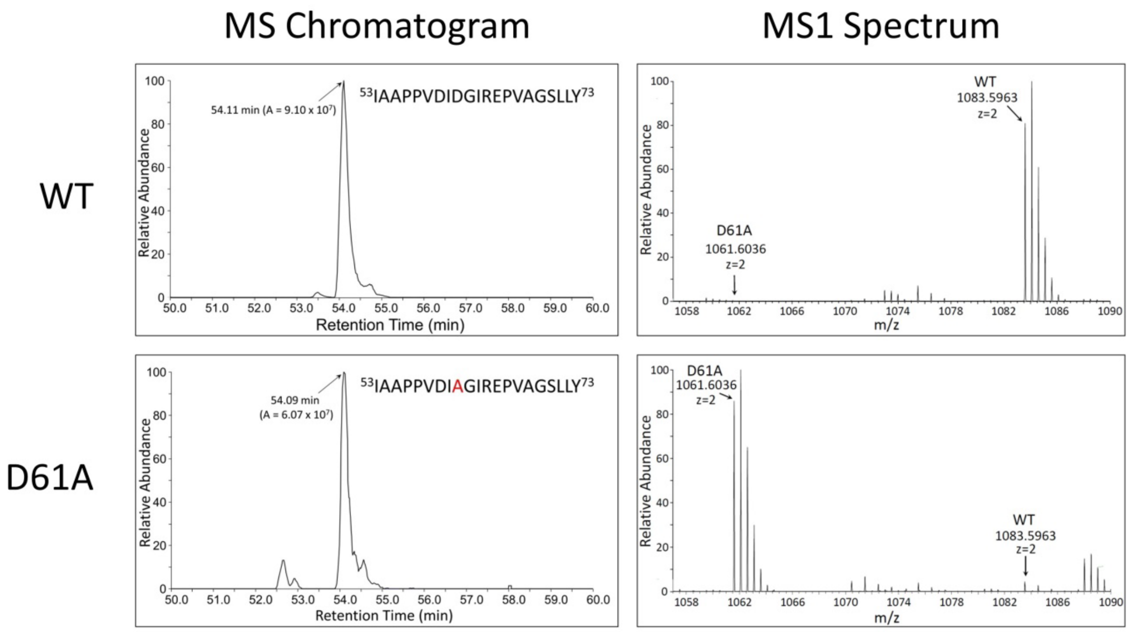

Supplementary Fig. 3 Comparison of the MS chromatograms and MS1 spectra of the chymotryptic peptide purified from WT and D1-D61A PSII core complexes that contains position 61 of the D1 subunit. Approx. 98.5% of the peptide from D1-D61A contained the desired Ala residue (red). In the MS1 spectra, the difference between the WT peak at 1083.5963 (z = 2) and the D61A peak at 1061.6036 (z = 2) shows a mass decrease of 44 Da in the mutant as expected from changing Asp to Ala at position 61.

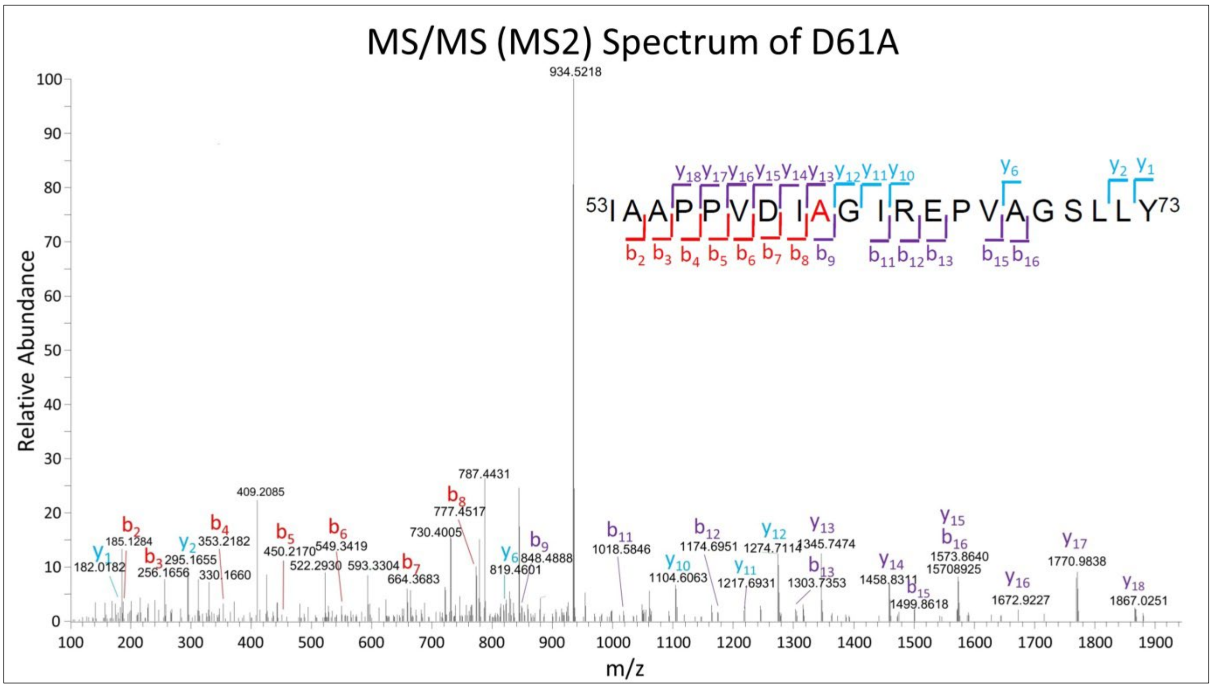

Supplementary Fig. 4 The MS/MS spectrum of the chymotryptic peptide purified from D1-D61A PSII core complexes that contains position 61 of the D1 subunit. The masses of the *y* fragments *y*18, *y*17, *y*16, *y*15, *y*14, and *y*13 (violet) and the *b* fragments *b*9, *b*11, *b*12, *b*13, *b*15 and *b*16 (violet) confirm the identity of the peptide and the presence of the D1-D61A mutation.

2. Additional information on the O_2 polarography data.

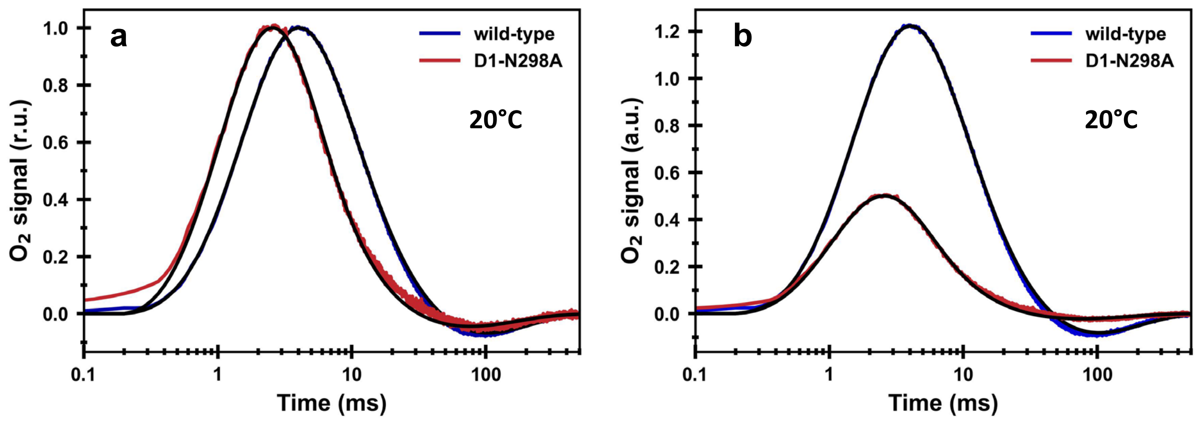

Supplementary Fig. 5 Direct comparison of O_2 polarography transients of wild-type and D1-N298A PSII thylakoid membranes at 20 °C. Transients (blue and red) and their fit curves (black) are shown **a** normalized to their peak and **b** with their original amplitudes. The data shown here is also shown in Fig. 2 of the article.

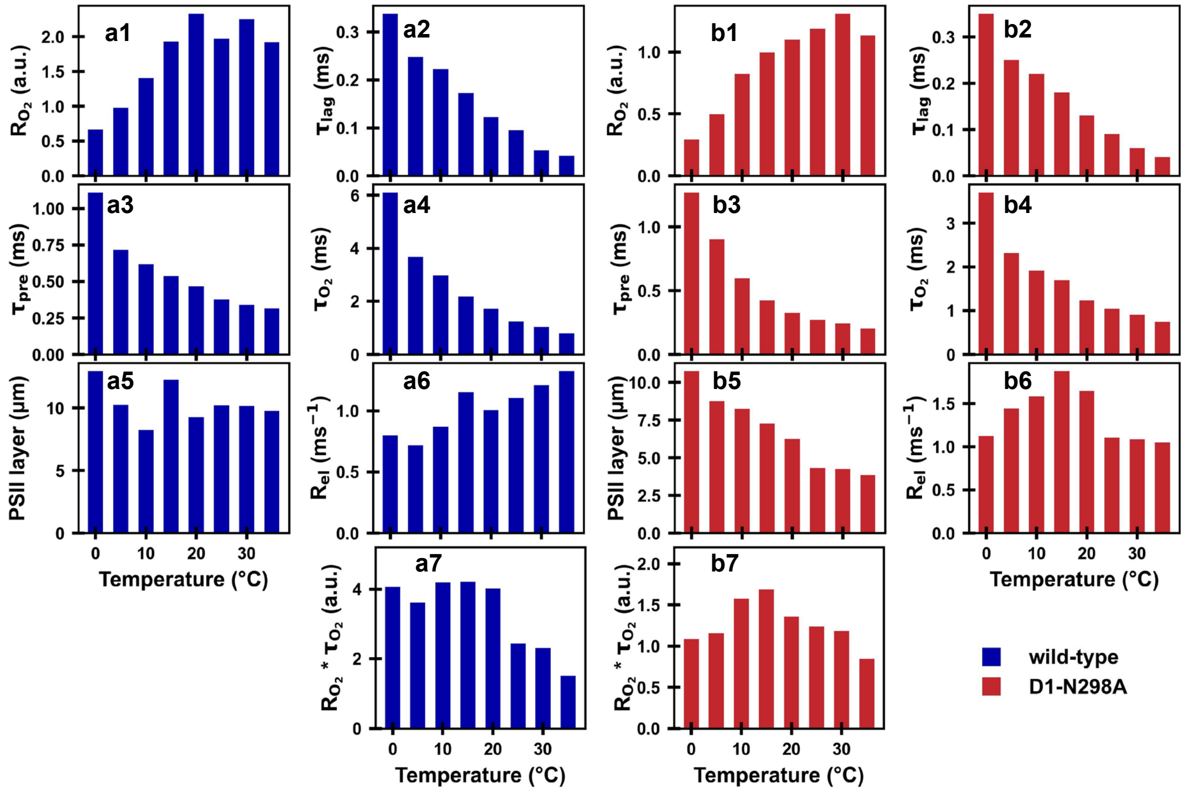

Supplementary Fig. 6 Parameters from fitting the O_2 polarography data to a diffusion model. The fit parameters are shown for all temperatures for **a** wild-type and **b** D1-N298A. **a1, b1** Parameter relating to the initial population of PSII in the S3 state. **a2, b2** Parameter addressing the delay of oxygen reduction by the electrode. **a3, b3** Time constant of the step preceding oxygen evolution (lag phase).

a4, b4 Time constant of the oxygen evolution reaction. **a5, b5** Parameter describing the thickness of the deposited PSII layer. **a6, b6** Oxygen reduction rate at the electrode. **a7, b7** Parameters showing a1 (or b1) and a4 (or b4) multiplied by each other. The product of the two parameters typically has its largest value around 10°C, but shows overall relatively little temperature dependence. The corresponding data is shown in Fig. 2 of the article; fit curves are shown in Supplementary Fig. 5 for 20°C.

Supplementary Table 1 Results from applying Arrhenius and Eyring analysis to the O_2-polarography data corresponding to phase preceding oxygen evolution step of wild-type PSII and PSII carrying the N298A mutation. The natural logarithm of the pre-factor A, as well as the activation energy E_a, were obtained from the Arrhenius plot in Fig.2c. From those values, the enthalpy ($\Delta H^\ddagger$), entropy ($-T_o\Delta S^\ddagger$) and Gibbs free energy of activation ($\Delta G^\ddagger$) were calculated using Supplementary Equation Set 1, with T_0 = 20°C. The time constant of phase preceding oxygen evolution at 20°C (τ_{pre}) is also shown.

Synechocystis sp. PCC 6803 variant	τ_{pre} at 20°C (ms)	ln(A) (s^{-1})	E_a (meV)	$\Delta H^\ddagger$ (meV)	$-T_o\Delta S^\ddagger$ (meV)	$\Delta G^\ddagger$ (meV)
**wild-type**	0.47 ± 0.09	18.3 ± 1.6	269 ± 25	244 ± 25	306 ± 30	550 ± 5
**N298A**	0.33 ± 0.06	23.9 ± 1.4	405 ± 24	380 ± 24	164 ± 29	541 ± 5

Supplementary Equations

The time constants were subjected to Arrhenius analysis, allowing for the determination of an activation energy (E_a) and pre-exponential factor (A) by linear regression (Fig. 2c of the article). The enthalpy and entropy of activation ($\Delta H^\ddagger$ and $\Delta S^\ddagger$) were calculated from E_a and A using the following set of equations (k_B, Boltzmann constant; h, Planck constant, T, absolute temperature in Kelvin; T_0, here 293,15 K corresponding to 20°C):

$$\Delta H^\ddagger = E_a - k_B T_o$$

$$\Delta S^\ddagger = k_B \ln\left(\frac{hA}{k_B T}\right) - k_B$$

$$\Delta G^\ddagger = \Delta H^\ddagger - T_o \Delta S^\ddagger$$

3. Static FTIR difference spectra

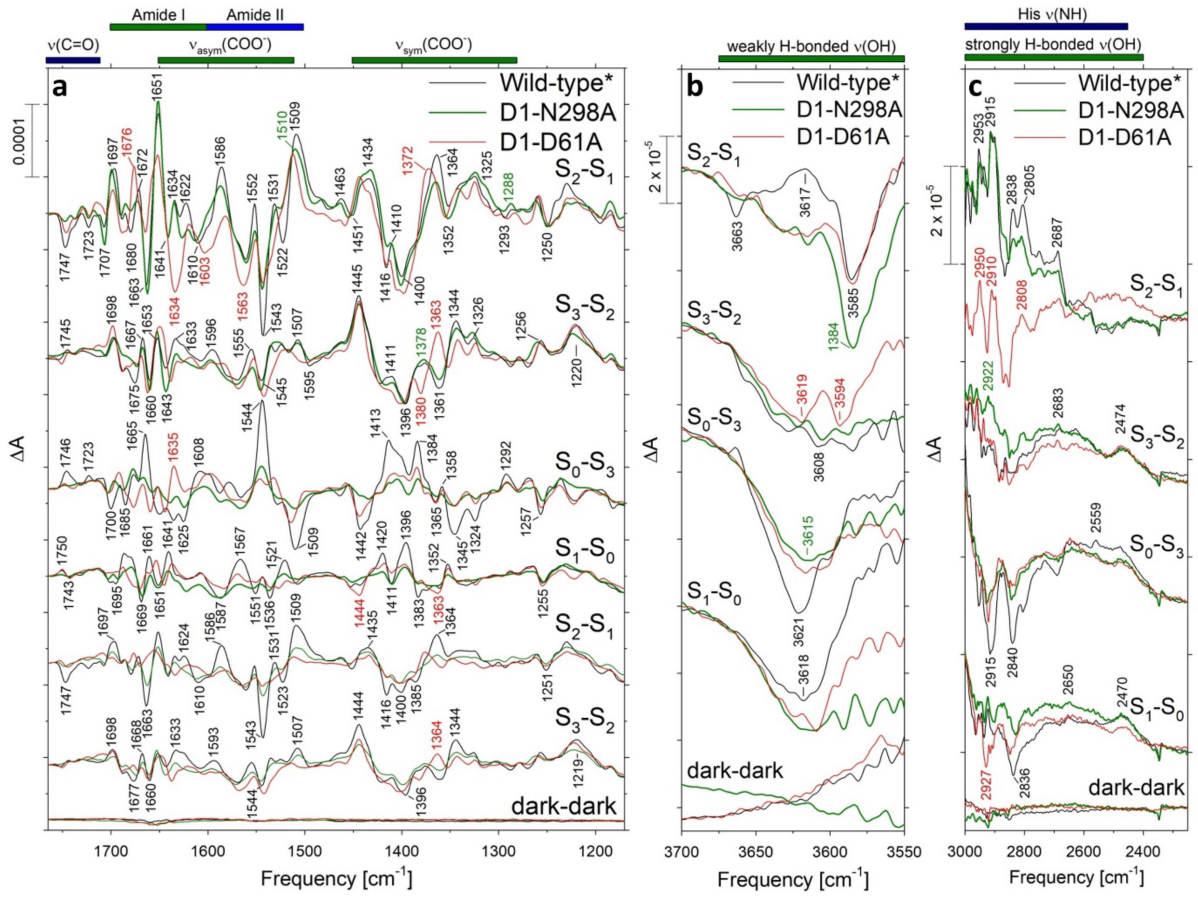

Supplementary Fig. 7 Static FTIR difference spectra of wild-type (black), D1-N298A (green) and D1-D61A (red) PSII core complexes in response to flash illuminations applied at 0°C. The wild-type data represent the averages of 22 samples (33,000 scans of 127 ms duration for each trace). The D1-N298A data represent the averages of 25 samples (37,700 scans for each trace). The D1-D61A data are replotted from ref. [1] and represent the averages of 16 samples (24,000 scans for each trace). For each flash, the spectra were normalized to the extent of the flash-induced formation of $Q_A^{\bullet-}$ as determined from the peak-to-peak amplitudes of the positive ferrocyanide band at 2038 cm^{-1} and the negative ferricyanide band at 2115 cm^{-1}. The data in panels **b** and **c** were collected simultaneously with those shown in panel **a**. Dark-dark control traces show the noise level and the stability of the baseline (lower traces in each panel). The type of vibrations visible in the respective spectral range is indicated on top of each panel; we note that for coordination to metal ions, the asymmetric (ν_{asym}) and symmetric (ν_{sym}) vibrations of carboxylate sidechains can cover a clearly broader spectral range than here indicated.

Notably, even though the N298A variation does not cause major changes in the S_1->S_2 and the S_2->S_3 difference spectra (Supplementary Fig. 7, *green*), some minor changes are detectable. These resemble alterations produced by the mutations of many other residues in the protein-water environment near the Mn_4Ca-oxo cluster, in particular the elimination of the ν(C=O) mode at 1747(-) cm^{-1}, the elimination of the amide II modes at 1531(+)/1522(-) cm^{-1}, the decreased amplitude of the $\nu_{asym}(COO^-)$ mode at 1586 cm^{-1}, the decreased amplitudes of the amide II modes at 1552(+) cm^{-1} and 1543(-) cm^{-1}, and the

decreased amplitude of the $\nu_{asym}(COO^-)$/amide II feature at 1509 cm^{-1}. For both genetic variants, the S-state difference spectra are reduced in amplitude at the 3rd and later flashes even though these are normalized according to the electrons reaching the artificial electron acceptor (ferricyanide) at the PSII acceptor side. The reasons for the decreased amplitudes are still unknown but may relate to side reactions at the PSII donor side in the relatively long interval between light flashes of 13 s (at 0°C) that was employed to obtain the spectra. Occasionally we have observed a similar effect also in PSII core complex preparations of wild-type PSII.

4. Time-resolved IR:

Experimental details, fit results, additional data, Supplementary Discussion

Supplementary Table 2 Number of averaged flash sequences per data set. At each wavenumber, data was acquired and analysed for 496 and 4670 flashes sequence. The specific number of repeats per wavenumber and PSII variant is given here.

	1384 cm^{-1}	1400 cm^{-1}	1514 cm^{-1}	1544 cm^{-1}
**wild-type**	2594	2699	3914	3276
**D61A**	1324	2377	2218	496
**N298A**	2507	4670	2086	1970

Supplementary Table 3 Time constants and amplitudes obtained from multiple global fits of the deconvolved IR data of the S_3->S_0 transition (Fig. 4a-d). The data was simulated (least-square fit) with a sum of 5 exponentials and an offset (Equ. 1 in Methods section of the article)**.** Fitting was performed on all possible subsets of the four transients (see text below); the given values are the averaged time constants and amplitudes along with their standard deviation. The amplitudes correspond to the bar plots in Fig. 4e-h.

	τ_1 (μs)	τ_2 (μs)	τ_3 (μs)	τ_4 (ms)	τ_5 (ms)
**wild-type**	6.2 ± 2.4	46.4 ± 18.4	479.9 ± 122.4	4.7 ± 0.3	59.6 ± 34.9
**D61A**	8.8 ± 2.2	110.5 ± 78.4	987.4 ± 368.9	15.3 ± 7.3	356.9 ± 57.3
**N298A**	5.5 ± 2.5	69.4 ± 33.4	788.1 ± 219.1	6.5 ± 0.8	74.6 ± 8.2

ν (cm^{-1})	A_1 (μoD)	A_2 (μoD)	A_3 (μoD)	A_4 (μoD)	A_5 (μoD)
			**wild-type**		
**1384**	-37.4 ± 1.9	-34.6 ± 1.9	-20.1 ± 1.3	60.2 ± 1.7	-2.7 ± 0.9
**1400**	16.4 ± 6.5	-27.8 ± 5.6	-15.9 ± 3.3	21.6 ± 0.8	1.7 ± 0.5
**1514**	-22.9 ± 4.6	1.4 ± 4.6	-4.9 ± 0.4	-37.5 ± 1.3	-8.8 ± 1.0
**1544**	-77.9 ± 11.3	-44.1 ± 13.0	-28.7 ± 1.2	111.6 ± 2.2	-11.4 ± 2.1
			**D61A**		
**1384**	-18.1 ± 1.0	-19.4 ± 1.1	-17.4 ± 1.3	-10.3 ± 0.4	12.2 ± 0.7
**1400**	-7.5 ± 1.8	-11.8 ± 2.1	-13.1 ± 3.3	-6.7 ± 0.3	8.3 ± 0.6
**1514**	-15.2 ± 0.3	-3.2 ± 0.3	-6.0 ± 0.9	1.6 ± 0.3	-18.6 ± 0.2
**1544**	-41.1 ± 2.8	-18.4 ± 0.8	-17.8 ± 4.4	2.9 ± 0.5	20.4 ± 1.5
			**N298A**		
**1384**	-31.7 ± 4.3	-34.9 ± 2.8	-22.6 ± 2.2	17.6 ± 2.3	25.4 ± 0.7
**1400**	-9.4 ± 0.4	-24.2 ± 1.5	-8.2 ± 1.8	-2.2 ± 0.4	7.2 ± 0.1
**1514**	-39.8 ± 5.5	-7.3 ± 7.8	-21.7 ± 1.0	-16.1 ± 1.3	-27.9 ± 0.6
**1544**	-55.7 ± 3.8	-29.4 ± 5.6	-14.2 ± 2.5	31.1 ± 1.4	16.0 ± 0.5

The IR time constants of the S_3->S_0 transition were determined by globally fitting all possible subsets of the four transients (1384, 1400, 1514 and 1544 cm^{-1}) and then calculating the average value and its standard deviation. Some fits of the D61A and N298A variants deviated strongly from the mean and were treated as outliers (i.e. they were omitted). This resulted in 15 sets of fit parameters for wild-type PSII and 10 for both D61A and N298A. The averaged time constants and standard deviation are shown in Supplementary Table 3 (top); the corresponding averaged amplitudes and their standard deviation can be found in Supplementary Table 3 (bottom). The results of the individual fits for wild-type, D61A and N298A are visualized in Supplementary Figs. 8–10, respectively.

In Fig. 4e-h of the article, the averaged time constants and amplitudes are shown. In Fig. a-d, however, the fit curves corresponding to the global fits of all four wavenumbers are shown. The fit parameters corresponding directly to the shown fit curves are given in Supplementary Table 4. They are very similar to the averaged values (Supplementary Table 3).

Supplementary Table 4 Time constants and amplitudes obtained from globally fitting the deconvolved IR data of the S_3->S_0 transition (Fig. 4a-d) at four wavenumbers. The data was simulated (least-square fit) with a sum of 5 exponentials and an offset (Equ. 1 in Methods section of the article)**.** The fit results shown here directly correspond to the fit curves shown in Fig. 4a-d. Unlike in Supplementary Table 3, only a single global fit was performed per PSII variant. The given errors of the time constants are the fit uncertainties estimated by the least-squares minimization algorithm; they are clearly unrealistically small.

	τ_1 (µs)	τ_2 (µs)	τ_3 (µs)	τ_4 (ms)	τ_5 (ms)
**wild-type**	7.6 ± 0.1	54.5 ± 0.1	503.3 ± 0.9	4.7 ± 0.1	44.3 ± 0.1
**D61A**	8.0 ± 0.1	73.6 ± 0.4	804.1 ± 4.1	13.8 ± 0.1	381.4 ± 0.4
**N298A**	8.2 ± 0.1	103.3 ± 0.2	776.5 ± 1.5	6.8 ± 0.1	74.0 ± 0.1

ν (cm^{-1})	A_1 (µoD)	A_2 (µoD)	A_3 (µoD)	A_4 (µoD)	A_5 (µoD)
			**wild-type**		
**1384**	-36.8	-33.0	-21.5	60.4	-3.1
**1400**	14.1	-29.1	-12.9	21.8	1.5
**1514**	-20.9	4.7	-5.2	-36.4	-9.6
**1544**	-84.9	-36.1	-27.7	109.4	-9.5
			**D61A**		
**1384**	-18.4	-18.8	-18.3	-10.0	13.0
**1400**	-6.5	-10.6	-14.9	-6.5	7.7
**1514**	-15.3	-2.7	-6.4	1.7	-18.4
**1544**	-38.5	-17.1	-22.2	2.2	21.7
			**N298A**		
**1384**	-36.2	-31.1	-19.1	16.1	25.4
**1400**	-8.9	-23.7	-9.4	-2.1	7.1
**1514**	-37.3	-0.7	-22.4	-15.2	-28.0
**1544**	-59.3	-23.4	-10.8	30.0	15.3

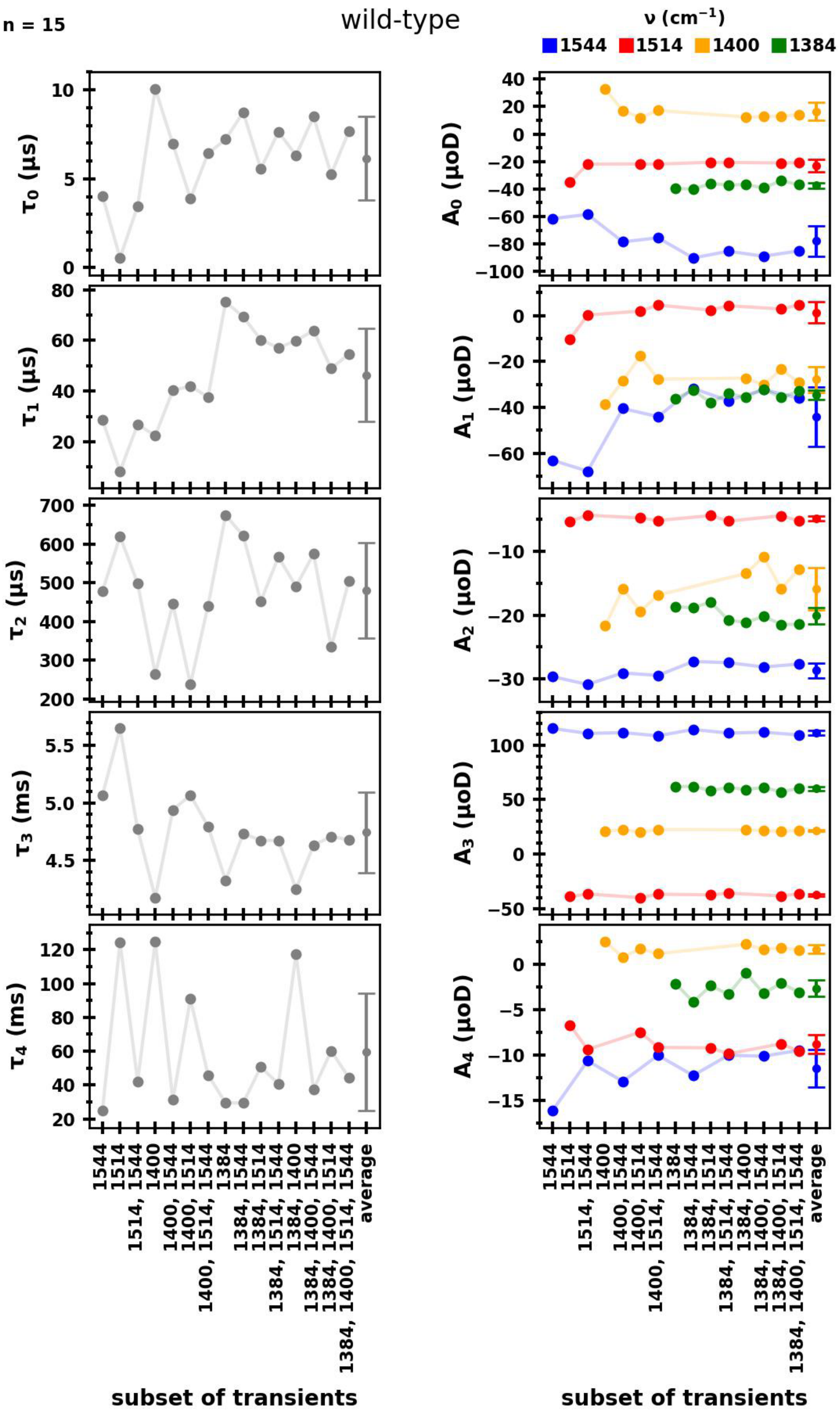

Supplementary Fig. 8 Error estimation for the parameters of the S_3->S_0 global fit of wild-type PSII. For every subset of the four analyzed transients (1384, 1400, 1514, and 1544 cm^{-1}), the data was globally fit to a sum of five exponentials and an offset (Equ. 1 in Methods section of the article). The time constants (left column) and corresponding amplitudes (right column) are shown for each individual fit. In each panel, the mean value is also shown along with an error bar indicating the standard deviation. The mean values and standard deviations are also given in Supplementary Table 3.

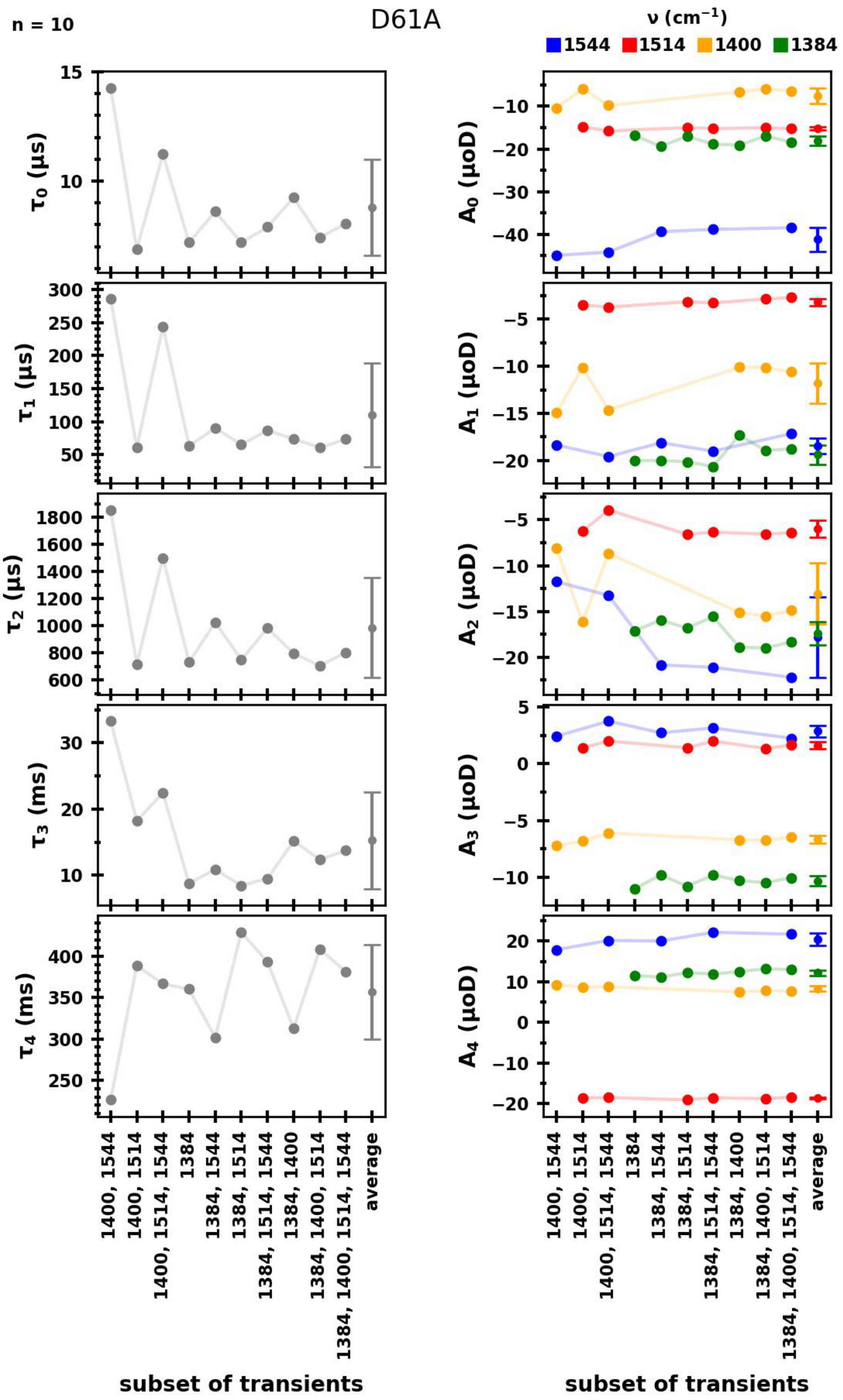

Supplementary Fig. 9 Error estimation for the parameters of the S_3->S_0 global fit of D61A. For every subset of the four analyzed transients (1384, 1400, 1514, and 1544 cm^{-1}), the data was globally fit to a sum of five exponentials and an offset (Equ. 1 in Methods section of the article). The time constants (left column) and corresponding amplitudes (right column) are shown for each individual fit. In each panel, the mean value is also shown along with an error bar indicating the standard deviation. The mean values and standard deviations are also given in Supplementary Table 3. The results of five subsets deviated strongly and were treated as outliers (omitted here).

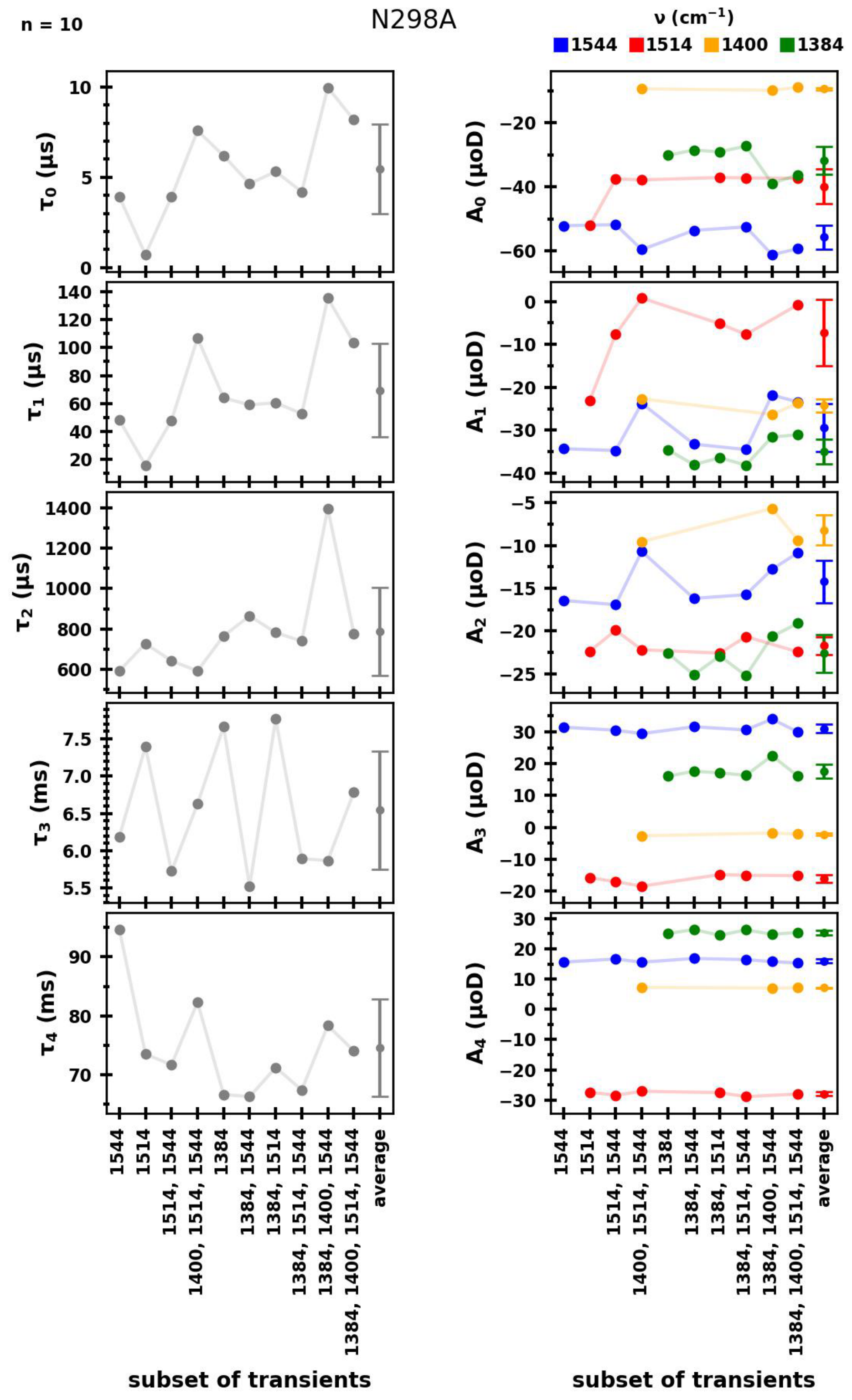

Supplementary Fig. 10 Error estimation for the parameters of the S_3->S_0 global fit of N298A. For every possible subset of the four analyzed transients (1384, 1400, 1514, and 1544 cm^{-1}), the data was globally fit to a sum of five exponentials and an offset (Equ. 1 in Methods section of the article). The time constants (left column) and corresponding amplitudes (right column) are shown for each individual fit. In each panel, the mean value is also shown along with an error bar indicating the standard deviation. The mean values and standard deviations are also given in Supplementary Table 3. The results of five subsets deviated strongly and were treated as outliers (omitted here).

Selected transients of the 1st and 2nd flash data (predominantly S_1->S_2 and S_2->S_3, respectively) are shown in Supplementary Fig. 11. The time constants shown in panels b and d were obtained by fitting all four transients (1384, 1400, 1514 and 1544 cm^{-1}) globally to a sum of exponentials (unlike for the S_3->S_0 data, no fitting of subsets was performed). For the S_1->S_2 transition, the results suggest a comparatively minor slow-down of the ET step from about 100 µs to 170 µs in the N298A mutant, but a major slowdown to about 530 µs in the D61A variant. In the S_2->S_3 transition, both mutants (especially D16A) show non-negligible slow millisecond contributions, which is nearly absent in the wild-type data. Compared to the 3rd flash data, however, the kinetics of the 1st and 2nd flash data are overall poorly resolved in most of the four measured transients. Further experiments will be necessary to allow for an in-depth analysis and interpretation of the effect of the N298A and D61A mutations on the S_1->S_2 and S_2->S_3 transitions.

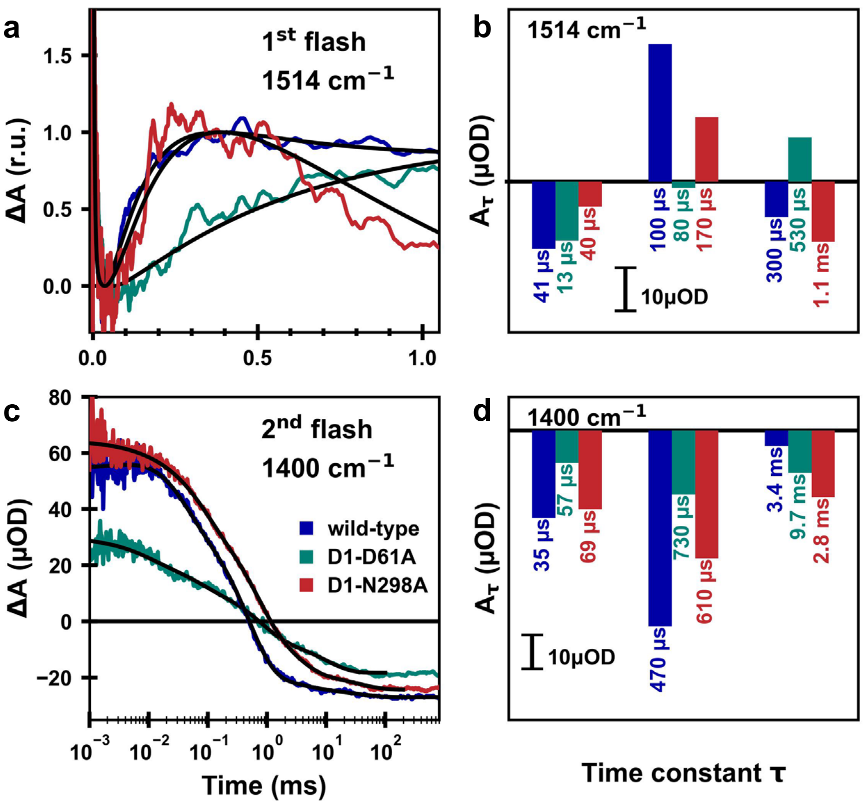

Supplementary Fig. 11 Time-resolved IR difference data of the 1st and 2nd flash data of PSII core particles from *Synechocystis* sp. PCC 6803. a IR difference signal at 1514 cm^{-1} acquired after applying a single saturating excitation flash to the sample, which induces predominantly the S_1->S_2 transition. **b** Parameters obtained by fitting the data in **a** to a sum of exponentials (Equ. 1 in Methods section of the article). **c** IR difference signal at 1400 cm^{-1} acquired after applying two saturating excitation flashes to the sample, inducing mostly the S_2->S_3 transition. **d** Parameters obtained by fitting the data in **c** to a sum of exponentials. In **a-d** the wild-type data is shown in blue, D1-D16A in green, and D1-N298A in red.

Supplementary Fig. 12 shows the non-deconvolved 3rd flash data, analyzed analogously to the S_3->S_0 data shown in Fig. 4 of the article. While the time constants differ slightly from those determined for the S_3->S_0 data, the overall trends are the same.

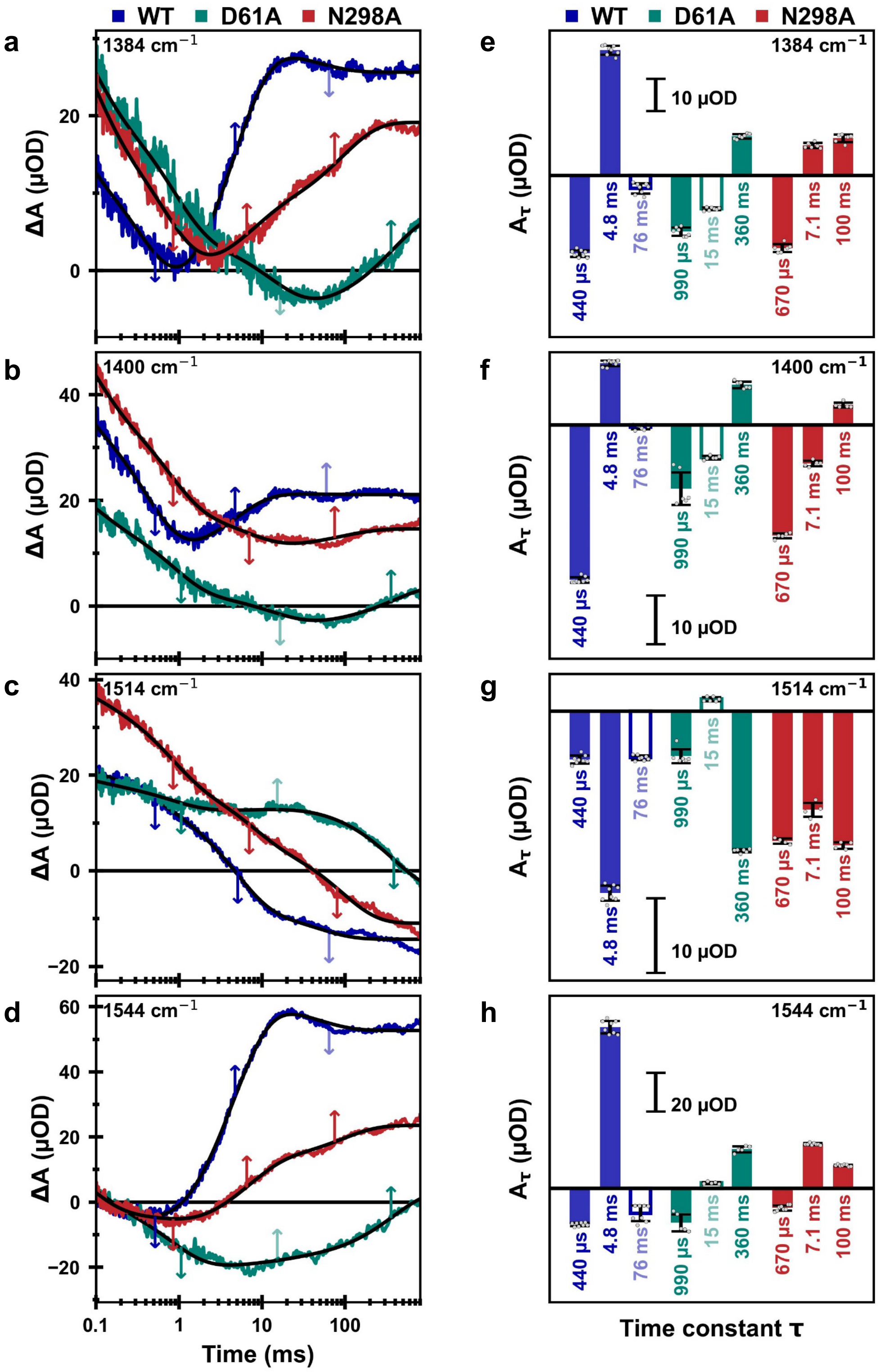

Supplementary Fig. 12 Time-resolved IR difference data of the 3rd flash data of PSII core particles from *Synechocystis* sp. PCC 6803. a-d IR difference signal acquired after applying a third saturating

excitation flash to the sample. The wild-type data is shown in blue, D1-D16A in red and D1-N298A in green at **a** 1384 cm^{-1}, **b** 1400 cm^{-1}, **c** 1514 cm^{-1} and **d** 1544 cm^{-1}; fit curves are shown in black. **e-h** Bar plots of the amplitudes corresponding to the time constants obtained from fitting the data on the left to a sum of exponentials. As opposed to Figure 4 of the article, the 3rd flash transients were analysed without applying a deconvolution procedure to obtain the transients of the pure S-state transitions; otherwise, the data analysis approach was the same. The bars filled in white are assigned to processes that are not specific for the oxygen-evolving S_3->S_0 transitions, see Supplementary Figs. 15 and 16. The time constants were determined globally by a joint fit of transients collected at several wavenumbers with the same set of time constants. Uncertainty ranges were estimated by performing fits of various subsets of the four transients (WT: n=15; N298A: n=10; D61A: n=10), for which the mean values of the time constants and amplitude are shown here, with the error bars indicating the respective standard deviations of the amplitudes. The amplitudes of the individual fits are additionally shown as grey dots. The time constant values are indicated by arrows in panels a-d.

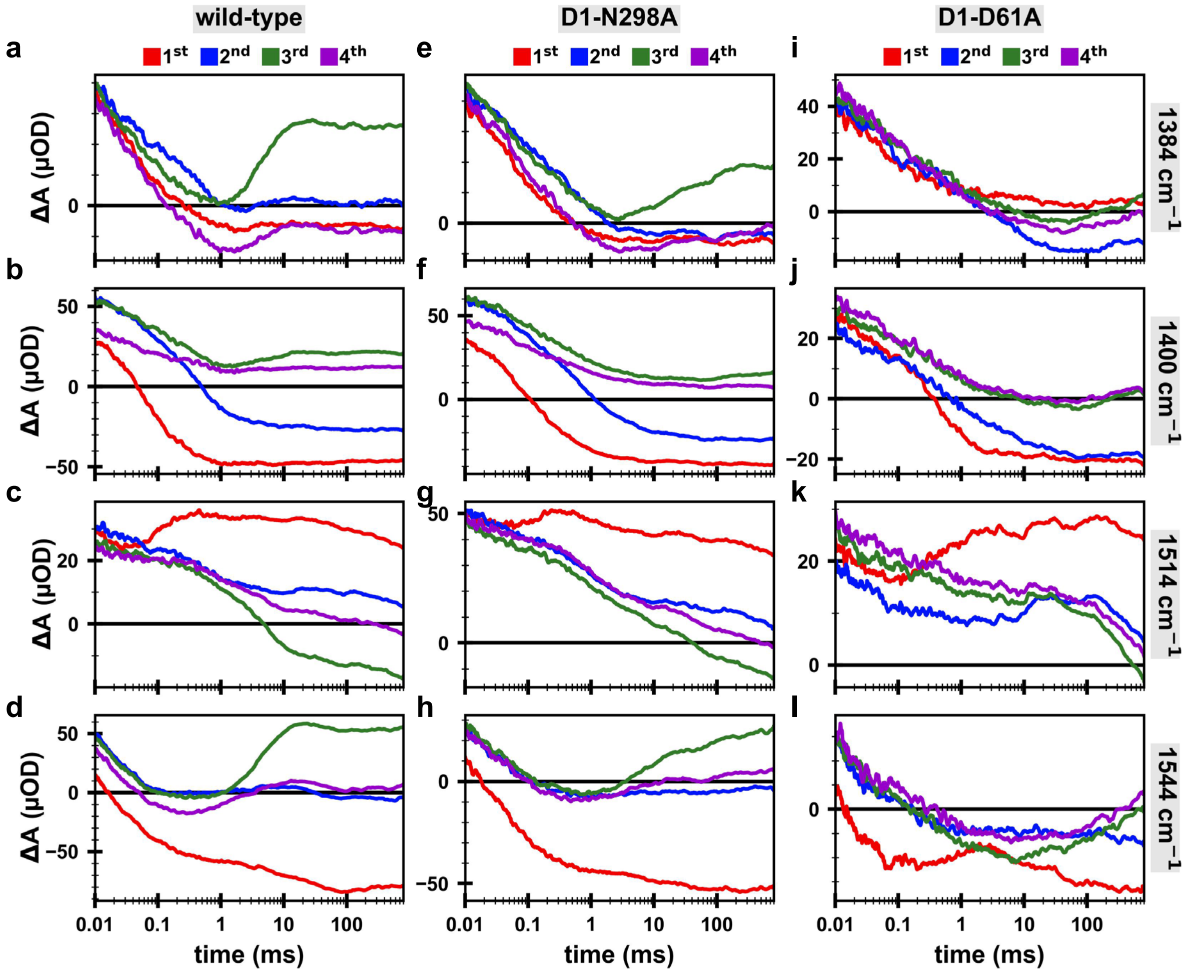

Supplementary Fig. 13 IR difference transients following the first four excitation flashes of PSII core particles from *Synechocystis* sp. PCC 6803. The 1st flash data (mostly S_1->S_2) is shown in red, the 2nd flash (mostly S_2->S_3) in blue, the 3rd flash (mostly S_3->S_0) in green, and the 4th flash in purple (mostly S_0->S_1) for **a-d** wild-type, **e-h** N298A and **i-l** D61A. For each PSII variant, the data is shown at 1384, 1400, 1514 and 1544 cm^{-1} (from top to bottom).

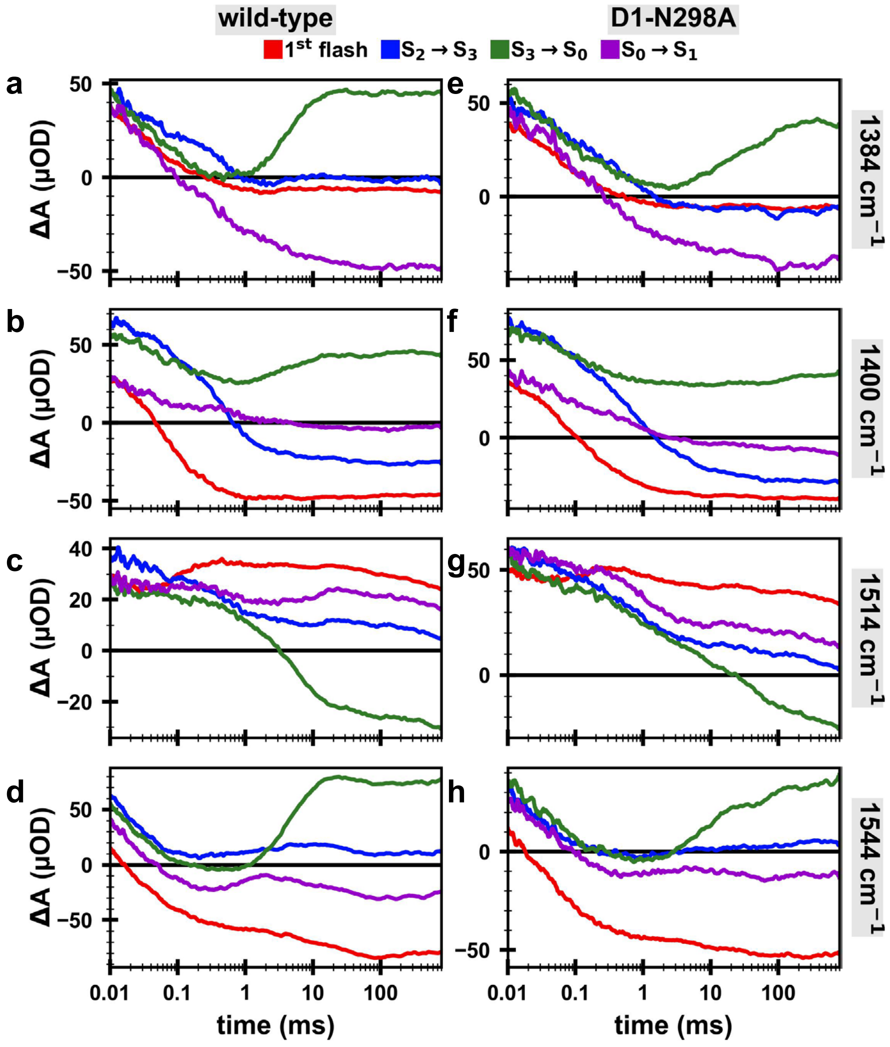

Supplementary Fig. 14 Deconvolved IR difference transients corresponding to the 'pure' S-state transitions. **a-d** Wild-type PSII and **e-h** PSII carrying the N298A at 1384, 1400, 1514 and 1544 cm^{-1} (top to bottom). The data was deconvolved under omission of the 1st flash data to obtain hypothetical transients of the pure S-state transitions; only for the S_1 transition the non-deconvolved first flash data is shown. For details of the deconvolution procedure, see the Methods section of the article.

Supplementary Discussion – Unassigned millisecond phases in wild-type and D61A

In the 3rd flash D61A IR transients a 15 ms phase was found (see Fig. 4), for which we assume that it is not assignable to the oxygen-evolving S_3->S_0 transitions, as justified in the following. In Supplementary Fig. 16a we show that the 15 ms phase is overall more strongly pronounced in the S_2->S_3 transition, indicating that this phase is not specific to the S_3->S_0 transition. This is also evident when comparing the millisecond regime of the 2nd and 3rd flash transients by visual inspection of Supplementary Fig. 15: Around 15 ms (highlighted in blue) a strong decay is present in the S_2 transition at 1384 cm^{-1} and 1400 cm^{-1} (panels a and b), which is less pronounced in the S_3 transition. In contrast, there is a strong rise in the hundreds of milliseconds (highlighted in green), which is clearly most pronounced in the 3rd flash data (and also in the 4th flash data, which is expected due to the poor S-state cycling efficiency of the D61A variant.

In the S_3->S_0 transition of wild-type PSII a 60 ms phase was found (Fig. 4). A similar phase is present in both the 2nd and 3rd flash transients (Supplementary Fig. 16b). Supplementary Fig. 17 a,d demonstrates that a similar phase is also prominent at 1478 cm^{-1}, where Q_A^- and Q_B^- strongly absorb. We thus conclude that the 60 ms phase is not specific for the S_3->S_0 transition and likely assignable to an acceptor-side event. Figure S18 shows that the biphasicity of the IR transients of the oxyen-evolving S_3->S_0 transition of the N298A PSII is clearly resolvable and unrelated to the 60 ms phase.

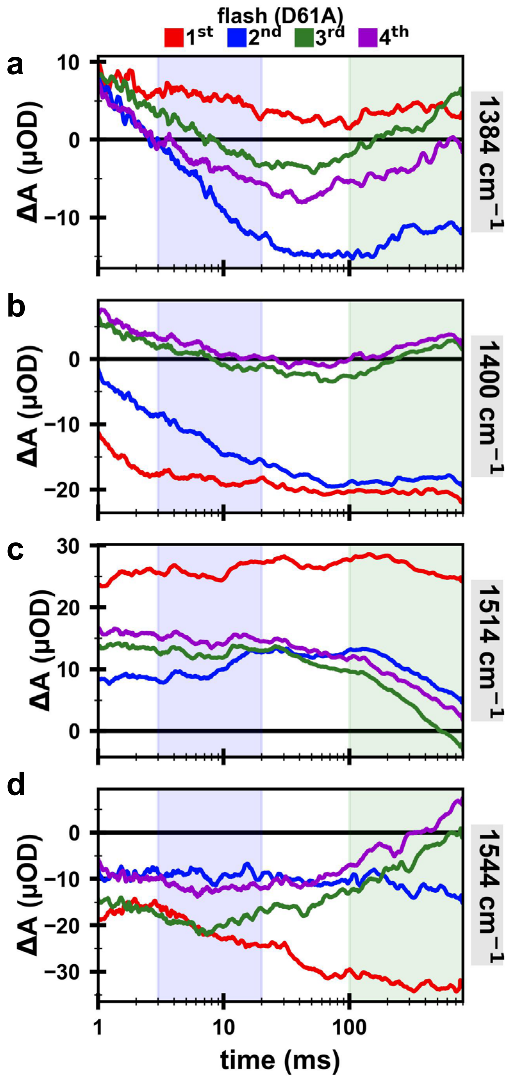

Supplementary Fig. 15 Infrared absorption changes in the millisecond region of the D61A mutant. IR changes are shown for the first four excitation flashes at **a** 1384 cm^{-1} **b** 1400 cm^{-1} **c** 1514 cm^{-1} and **d** 1544 cm^{-1}. The kinetics at around 15 ms (shaded blue area) are not specific to the S_3->S_0 transition (3rd flash, green lines) – in fact they are more pronounced in the S_2->S_3 transition (2nd flash, blue lines). In contrast, the kinetics around 360 ms (shaded green area) are clearly most pronounced in the S_3->S_0 transition (3rd flash, green lines). Note that the 4th flash data looks very similar to 3rd flash data, explainable by the poor S-state cycling efficiency of the D61A mutant.

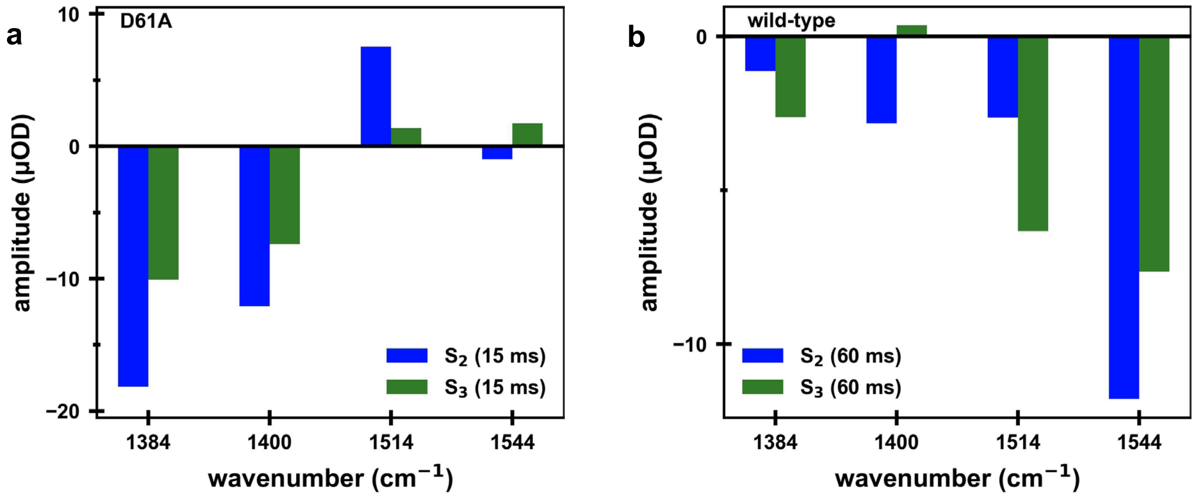

Supplementary Fig. 16 Comparative analysis of the millisecond region of the 2nd flash (mostly S_2->S_3, blue bars) and 3rd flash (mostly S_3->S_0, green bars) IR transients. The transients of **a** D61A and **b** wild-type were fit between 2-800 ms to a sum of two exponentials. For D61A, one of the time constants was fixed to 15 ms; for wild-type one time constant was fixed to 60 ms. The resulting amplitudes of these phases in the S_2->S_3 (blue) and the S_3->S_0 transition (green) are shown for the IR transients collected at 1384, 1400, 1514 and 1544 cm-1.

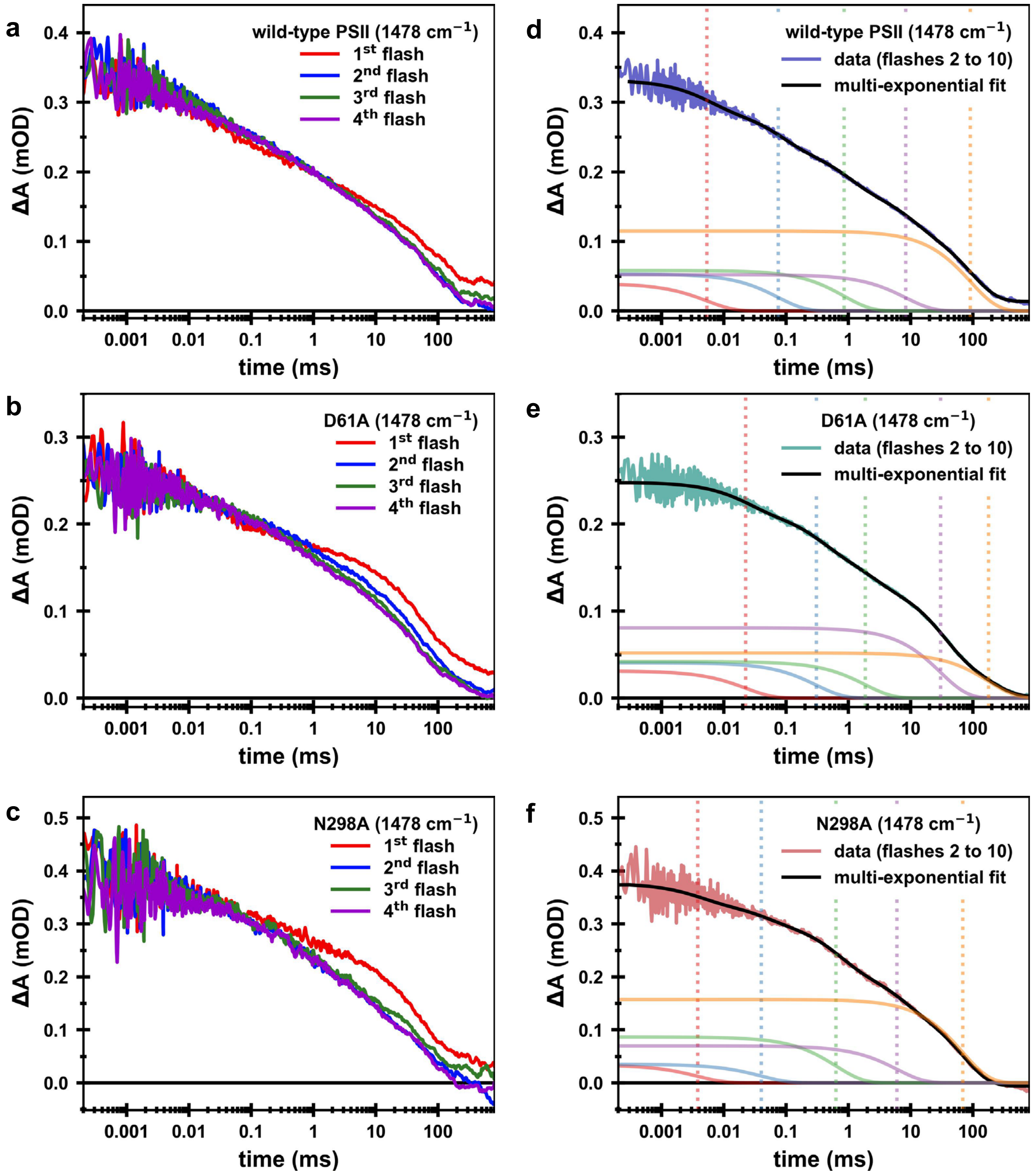

Supplementary Fig. 17 IR absorption changes at 1478 cm^{-1} for the three PSII variants. a-c IR difference absorption following the first four excitation flashes for **a** wild-type **b** D61A and **c** N298A. In panels **d-f** the transients following the 2nd to 10th excitation flash were averaged for better signal-to-noise and then fit to a sum of exponentials. The resulting fit curves are shown in black lines; the time constants are indicated by colored vertical lines. The individual single-exponential curves are shown in the same colors as the corresponding time constants. IR changes at 1478 cm^{-1} are predominantly associated with the quinones at the acceptor-side of PSII[2].

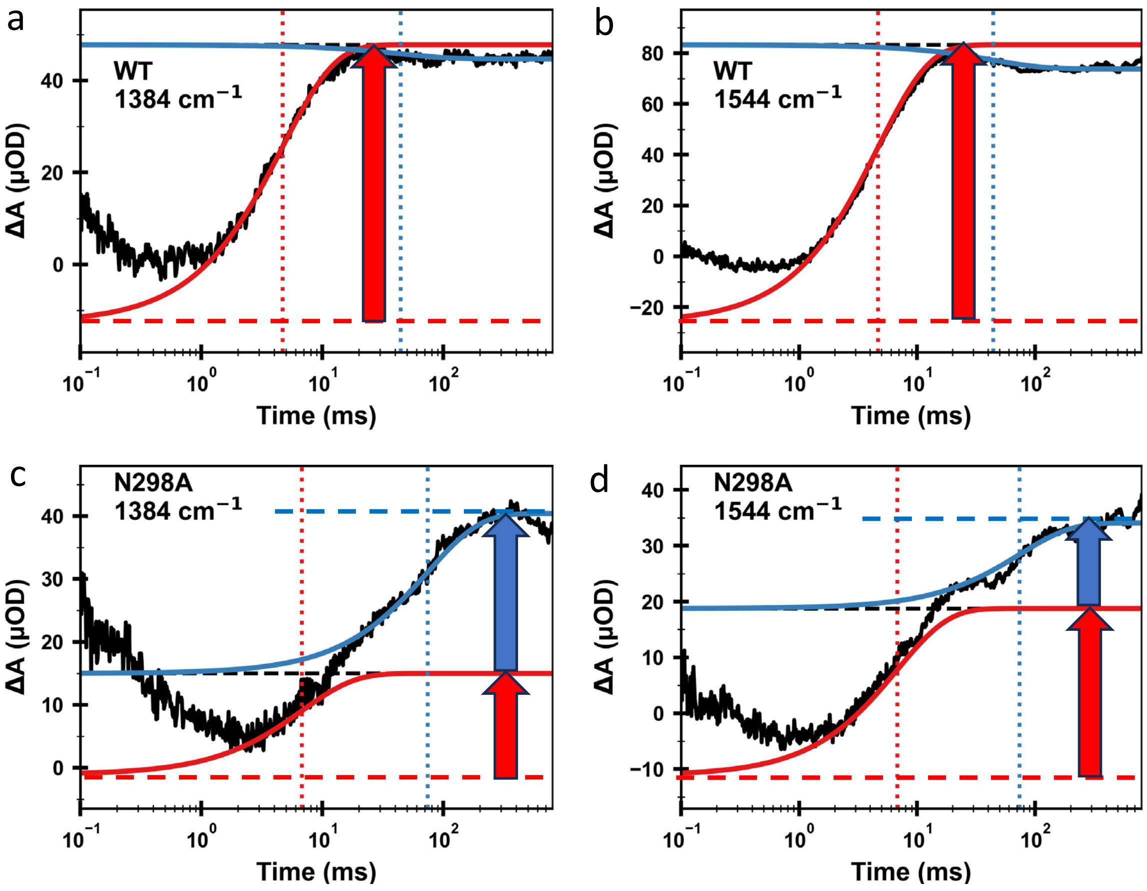

Supplementary Fig. 18 Millisecond rise of the IR transients in the S_3->S_0 transition for WT and N298A PSII. The IR transients and the fit curves correspond to the 1384 and 1544 cm^{-1} transients in Figure 4 of the article. The here used presentation mode illustrates how the monophasic rise in the WT PSII (red curves in a and b, 4.7 ms), which is assignable to the O_2-formation step, is replaced by a bi-phasic rise in the N298A variant (red curves in c and d, 6.5 ms; blue curves in c and d, 75 ms). In the WT transients, a minor 60 ms contribution is visible (blue curves in a and b), which is not assignable to a donor-side specific process of the S_3->S_0. The vertical dotted lines indicate the values of the respective time constants. Deviation between data and fit curves occasionally observed for times exceeding 400 ms could result from artefactual drift effects in the IR transients. We note that the ratio of the amplitudes of the two rise phases in N298A is not wavenumber-independent (see Figure 4 of the article for the amplitudes at 1400 and 1514 cm^{-1}), which is in line with the assignment to two distinct processes in the S_3->S_0 transition.

5. Molecular Dynamics

Detailed Methods

The initial structure of Photosystem II at 1.93 Å resolution was taken from the protein data bank (PDB ID: 7N8O)[3]. To model the $S_3Y_Z^{ox}$ state, the Mn_4CaO_6 cluster was taken from the crystallographically characterized PSII of *Thermosynechococcus vestitus* BP-1 (PDB ID: 8F4D)[4], together with the C-terminus of D1, D1-Ala 344, which is "poorly resolved, and therefore modeled with low confidence" in the available high-resolution cryo-EM structure for Synechocystis PSII[3]. Standard protein residues were described using the AMBER14SB force field[5], while force fields for β-carotene, chlorophyll a, pheophytin a, plastoquinone 9, digalactosyl diacylglycerol (DGDG), monogalactosyl diacylglycerol (MGDG), phosphatidylglycerol (PG) and sulfoquinovosyl diacylglycerol (SQDG) were developed as described in previous works[6,7]. The radical tyrosine (Y_Z) was described by the generalized Amber force field (GAFF)[8]. Optimization and electrostatic potential calculations were performed by using Gaussian 16[9] at the Hartree–Fock level with the 6–31G* basis set. Lennard-Jones parameters for the Mn_4CaO_6 cluster were taken in agreement with previous studies[6].

The parameters for bonds and angles of the Mn_4CaO_6 cluster, the three water/hydroxide molecules coordinated with Mn_4 and Mn_1 in the cryo-EM structure[3], and the side chain of the amino acids directly interacting with the Mn_4CaO_6 cluster (i.e. Asp170, Glu189, Glu333, Asp342, Ala344 and CP43-Glu341) were derived from QM/MM simulations of a previous work[10], following the approach proposed by Cárdenas and coworkers[11]. Dihedral terms were set to zero. The two water molecules coordinating the Ca ion (i.e. W3 and W4) were deliberately not constrained with bonds to the Mn_4Ca to sample the possible configurations adopted along the simulated trajectories. Notably, no constraints were applied to represent the Mn_4Ca-protein-water hydrogen bond network. To the best of our knowledge, the present study represents the first case in which the above-mentioned bond parameters of the Mn_4Ca cluster, and its neighbour ligands, have been assigned based on *ab initio* calculations.

Concerning the calculation of the partial charges associated with the Mn_4CaO_6 cluster, we considered the structure including the Mn_4CaO_6 cluster, the three water/hydroxide molecules coordinated with Mn4 and Mn1, and the side chain of the amino acids directly interacting with the Mn_4CaO_6 cluster, as previously done by Bovi et al.[7]. The structure was optimized at DFT level with the zora-def2-tzvpp basis set by using ORCA 5.0.1[12], constraining only the C_α atoms of the seven aforementioned amino acids. Then, the electrostatic potential was calculated on the optimized structure by using Gaussian16[9]. Both the structure optimization and the electrostatic potential calculation were performed in high-spin state using the unrestricted B3LYP density functional.

MD simulations were carried out using the GROMACS software package[13]. The protonation state of the histidines bound to heme molecules, or directly interacting with iron atoms or the magnesium atom in chlorophylls, was chosen according to their relative orientation in the cryo-EM structure. The other titratable residues were considered in their standard protonation state. The dimeric cryo-EM structure was embedded into a membrane bilayer consisting of 1594 POPC lipids described with Slipids/AMBER (FF) parameters[14].

The system was solvated in a box with dimensions 31.3 x 20.9 x 15.3 nm using the TIP3p water model[15]. Na^+ ions were used to neutralize the net negative charge of the simulated systems. The resulting system was first minimized with everything constrained except for the following: hydrogens, the D1-Ala 344 residue, the OEC, and the sidechains of its surrounding amino acids.

We then performed approximately 100 ns of MD simulation with harmonic position restraints (force constant 1000 kJ $mol^{-1}nm^{-2}$) on the heavy atoms of protein residues, cofactors and water molecules

present in the cryo-EM structure[3]. The surrounding amino acids of the OEC were constrained starting from their C_α atoms. The integration time step was gradually increased according to the following scheme:

- 1000 steps in NVT ensemble with a time step of 0.1 fs for numerical integration of the equations of motion.
- 10000 steps in NVT ensemble with a time step of 0.5 fs for numerical integration of the equations of motion.
- 10 ns in NVT ensemble with a time step of 1 fs for numerical integration of the equations of motion.
- 20 ns in NVT ensemble with a time step of 2fs for numerical integration of the equations of motion.
- 70 ns in NPT ensemble with a time step of 2fs for numerical integration of the equations of motion, using the Berendsen barostat[16].

Starting from the final structure of this run, the N298A point mutation was introduced. An additional 10 ns of position-restrained simulation was then run in parallel for both the wild-type PSII and the mutant. In the latter case, regenerated velocities were used, and the mutated residue was kept unrestrained.

Finally, 500 ns of unrestrained MD simulations were carried out in NPT ensemble. In total, ten classical molecular dynamics simulations were performed to investigate the wild-type protein and the N298A variant, five for each system with different starting velocities (namely WT-1, WT-2, WT-3, WT-4, WT-5, N298A-1, N298A-2, N298A-3, N298A-4, and N298A-5). Long-range electrostatic interactions were calculated using particle mesh Ewald method[17], using a grid spacing of 0.12 nm and a short-range cutoff of 1.2 nm. The LINCS algorithm[18] was applied to constrain the bond lengths of the hydrogen atoms to a constant value. A time step of 2 fs was used for numerical integration of the equations of motion. The temperature was kept constant by coupling the system using the V-rescale algorithm with a coupling time constant $\tau T = 0.1$ ps[19]. The system was also semi-isotropically coupled to a pressure bath at 1 bar with $\tau P = 1.0$ ps, using a Parrinello–Rahman barostat[20,21]. The simulated systems were composed of approximately 1,000,000 atoms each.

Cavity Volume Analysis

The cavity volume estimation was carried out using the Epock[22] plugin on VMD[23]. The Epock plugin requires a predefined volume defined by inclusion spheres around the mass center of a residue or around an atom, and from this volume the occupation of the protein is subtracted. To define the spatial bounds of the cavity, a combination of spherical volumes was used: one central sphere to include the region of interest, and one surrounding exclusion sphere to restrict it to the cavity only. The inclusion sphere of radius 6 Å was approximately centered on the water wheel cluster as already done to select contributing water molecules (i.e. by using the center of mass of C_α atoms of the residues D1-Val185, D1-His190, D1-Leu343, D1-Ala344, CP43-Ala386, and CP43-Ile398). To better capture the actual shape of the cavity, an additional exclusion sphere centered on the Mn4Ca cluster of radius 4.5 Å was used.

Overall stability

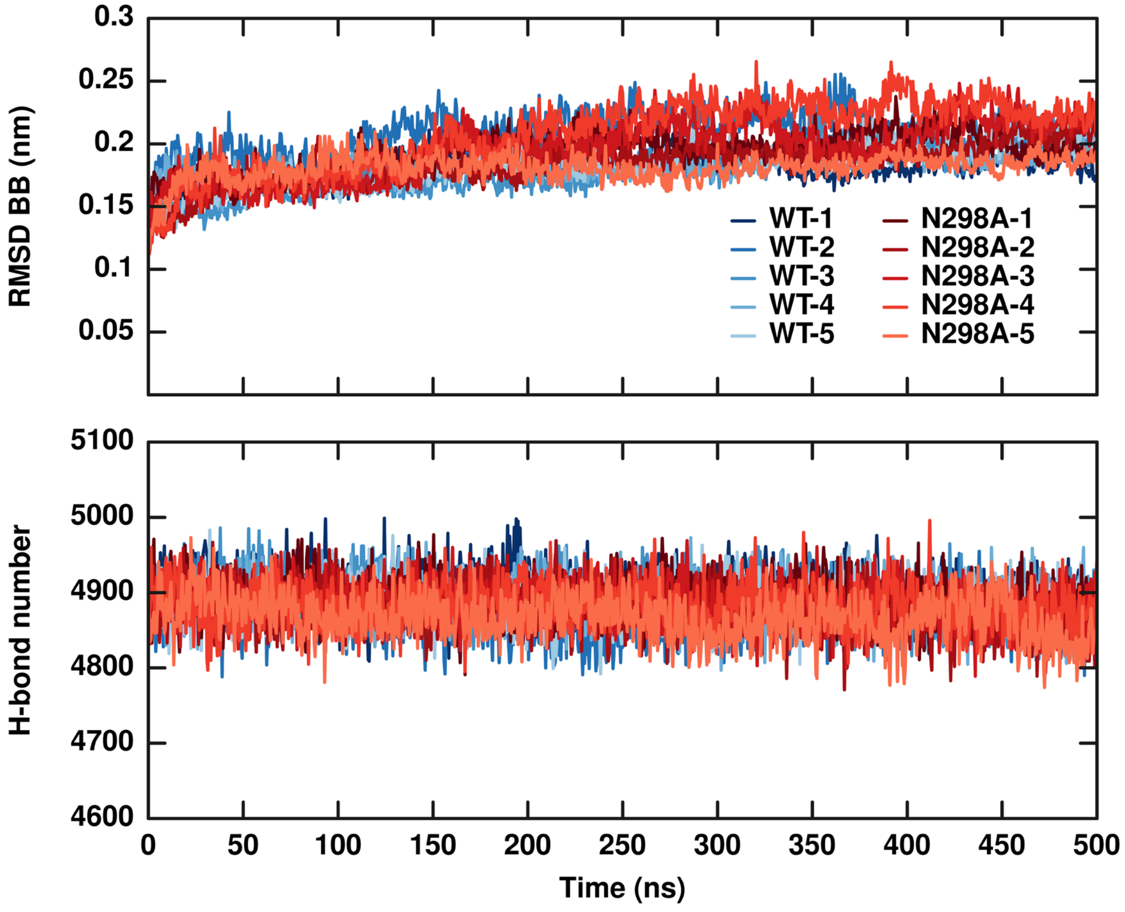

Supplementary Fig. 19 Overall stability of the simulated systems. Top panel: RMSD calculated on the protein backbone with respect to that of the starting structure (PDB ID: 7N8O) as a function of time for each simulated system. Bottom panel: number of H-bonds present in the whole protein as a function of time for each simulated system. The five WT replicas are shown in shades of blue; shades of red represent the five N298A replicas.

Water Wheel

Supplementary Table 5 Number of the contributing water molecules to the Water wheel. The table shows the average number of water molecules inside a shell of radius 3.8 Å and centered on the center of mass defined by the C_α atoms of the residues Val185, His190, Leu343, Ala344, CP43-Ala386, and CP43-Ile398. The data was collected over the last 400 ns of simulation for each of the 10 monomers per system (WT and N298A mutant). The standard error of the mean (SEM) was calculated using the average values from each monomer, which were treated as independent measurements.

Water Wheel	N. water molecules (SEM)
wild-type	5.0 (0.2)
N298A	4.5 (0.2)

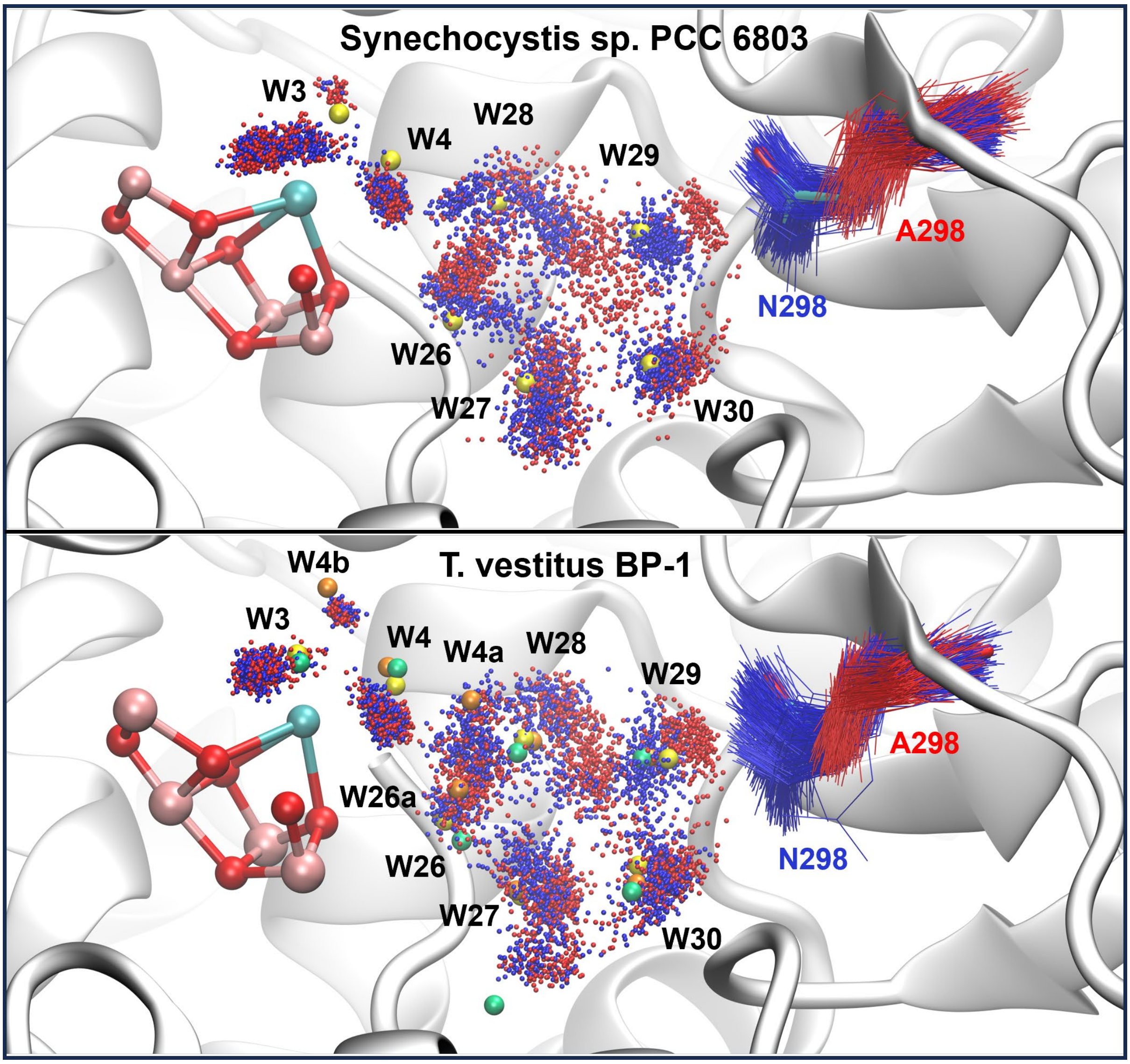

Supplementary Fig. 20 Water distribution in the target region for different cyanobacteria. The water distribution and MD conformations sampled for residue N298 (WT) and A298 (N298A mutant) using 40 snapshots from the last 400 ns of each of the ten trajectories are shown. Data was collected for both the mesophilic cyanobacterium, *Synechocystis* sp. PCC 6803, shown in the top panel, and *Thermosynechococcus vestitus* BP-1, shown in the bottom panel. Water molecules within a 3.8 Å sphere centered on the center of mass of the C_α atoms of the residues V185, H190, L343, A344, CP43-A386, and CP43-I398 (the last two corresponding to CP43-A399 and CP43-A411 in *T. vestitus*) were selected for the water wheel along with the water molecules inside a sphere of radius 3.0 Å centered on the calcium ion. In the top panel, the yellow spheres indicate the five waters of the pentagon together with W3 and W4 from the cryo-EM structure (PDB ID: 7N8O, from the mesophilic cyanobacterium, *Synechocystis* sp. PCC 6803)[3]. In the bottom panel the five waters of the pentagon together with W3 and W4 from the X-ray structure (PDB ID: 8F4D, PSII from *Thermosynechococcus vestitus* BP-1)[4] are represented by yellow (monomer 1) and green (monomer 2) spheres. Orange spheres show the positions of the water molecules as detected by the recent cryo-EM (PDB ID: 9EVX)[24]. Small blue and red spheres and lines refer to water molecules and residues from MD simulations of the wild-type and N298A variant, respectively. To ensure consistent comparison, all dynamic

fluctuations and structural snapshots—including those from *T. vestitus*—were aligned onto the Synechocystis cryo-EM structure by fitting the backbone atoms of D1-protein residues 165-210, 303-344 and residues 366-418 of the CP43 protein. Regarding the bottom panel (*T. vestitus*), the crystallographic model and our MD simulations relate to the $Y_Z^{ox}S_3$ state, whereas the cryo-EM structure was obtained for the PSII in its dark-stable S_1-state, which might indicate that the water-wheel structure is not strongly S-state dependent. The agreement between the structural and our computational results, including observation of transient population of the W4a and W4b sites, verifies the significance of our MD simulations.

Interaction distances and conformational variability of H190

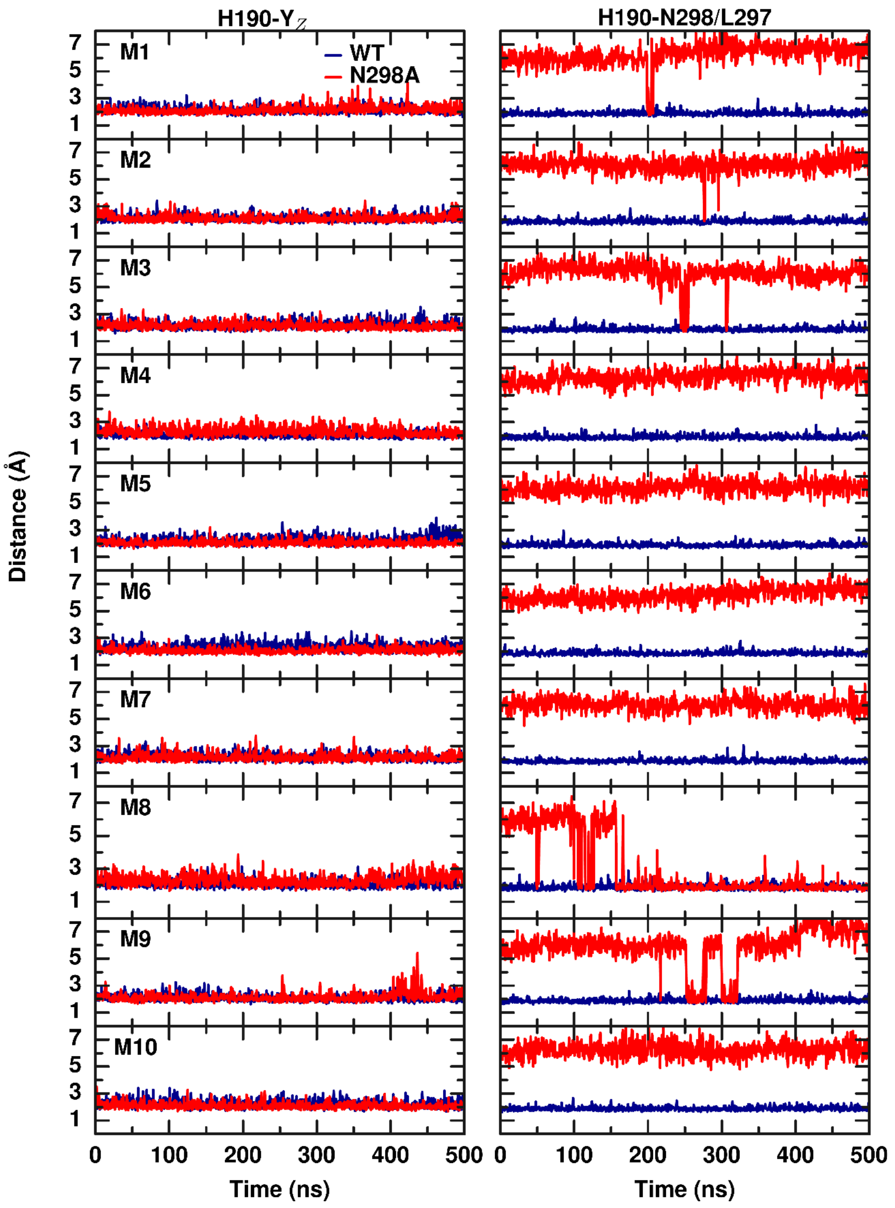

Supplementary Fig. 21 Y_z-H190 and H190-N298/L297 hydrogen bond distances calculated during the simulation time. From top to bottom, distances as a function of time from each monomer are shown.

Fluctuations for Y_z-H190 hydrogen bond are displayed on the left, while H190-N298/L297 fluctuations are shown on the right. The wild-type distances are plotted in blue, N298A distances in red.

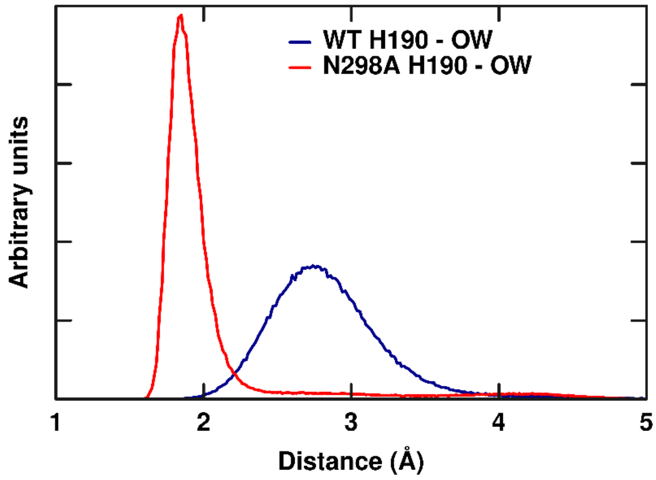

Supplementary Fig. 22 Distributions of distances of H190-Ow. The distribution of distances associated with the hydrogen bond to the delta nitrogen (N_δ) of H190 and water is shown. Histograms are created by considering the "abundance" of the respective sampled configurations, neglecting the first 100 ns of each trajectory (ten per system). The wild-type data are shown in blue, N298A in red.

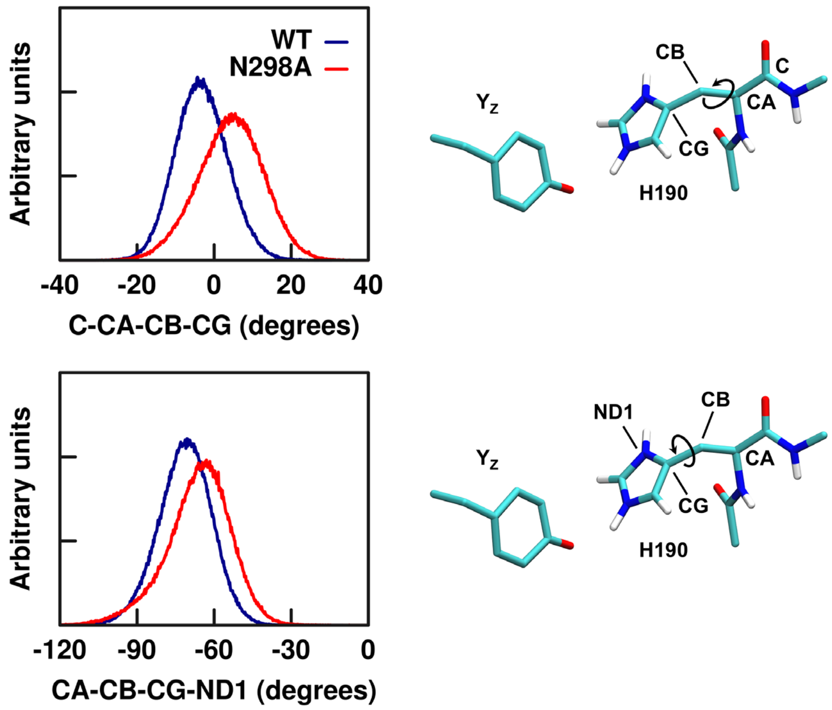

Supplementary Fig. 23 Comparison of dihedral angle distributions for the residue H190 in wild-type (WT, blue) and N298A mutant (red) systems. The panel on top shows the distribution of the C–CA–CB–CG dihedral angle, while the bottom panel shows the CA–CB–CG–ND1 dihedral angle. Both dihedrals describe sidechain conformations of H190. The right panels illustrate the corresponding

dihedral angles on the molecular structure. The shift in the distributions is consistent with a conformational rearrangement of H190 upon mutation of N298 to alanine. The broader distributions in the N298A mutant indicate increased conformational variability.

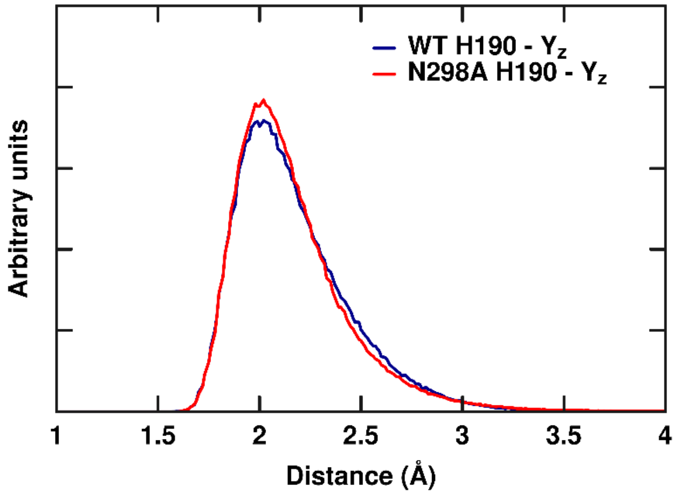

Supplementary Fig. 24 Distributions of distances of Y_Z (Y161)-H190. The distribution of distances associated with the Yz-H190 hydrogen bond is shown. Histograms are created by considering the "abundance" of the respective sampled configurations, neglecting the first 100 ns of each trajectory (ten per system). The wild-type data are shown in blue, N298A in red.

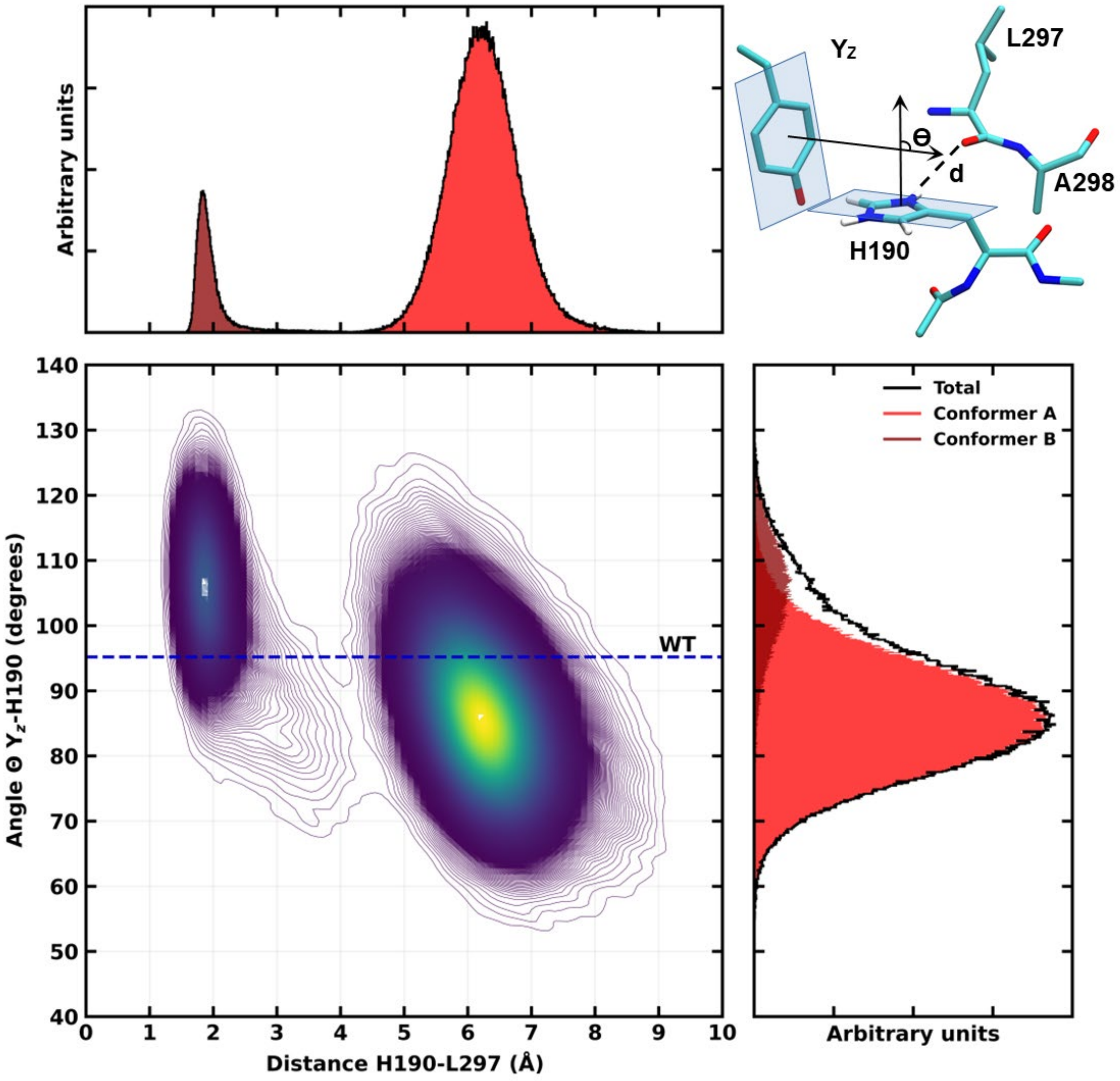

Supplementary Fig. 25 Two-dimensional distribution of the distance H190-L297 and the distribution of the angle Θ formed by the rings of Y_Z and H190 for the N28A mutant. The density map in the central panel highlights two distinct conformational states. The blue dashed line marks the average value observed for Θ in WT PSII. The top and right panels show the distributions of the H190-L297 distance and the Θ angle, respectively, with total (black), Conformer A (red), and Conformer B (dark red) contributions. The schematic representation in the top-right corner illustrates the geometric definition of Θ and the inter-residue distance used in the analysis. The plot shows how the two conformers of H190-L297 influence the relative orientation between the planes of Y_Z and H190. In WT PSII only one conformer is present, stabilized by the strong H-bond H190-N298 centered at 1.8 Å.

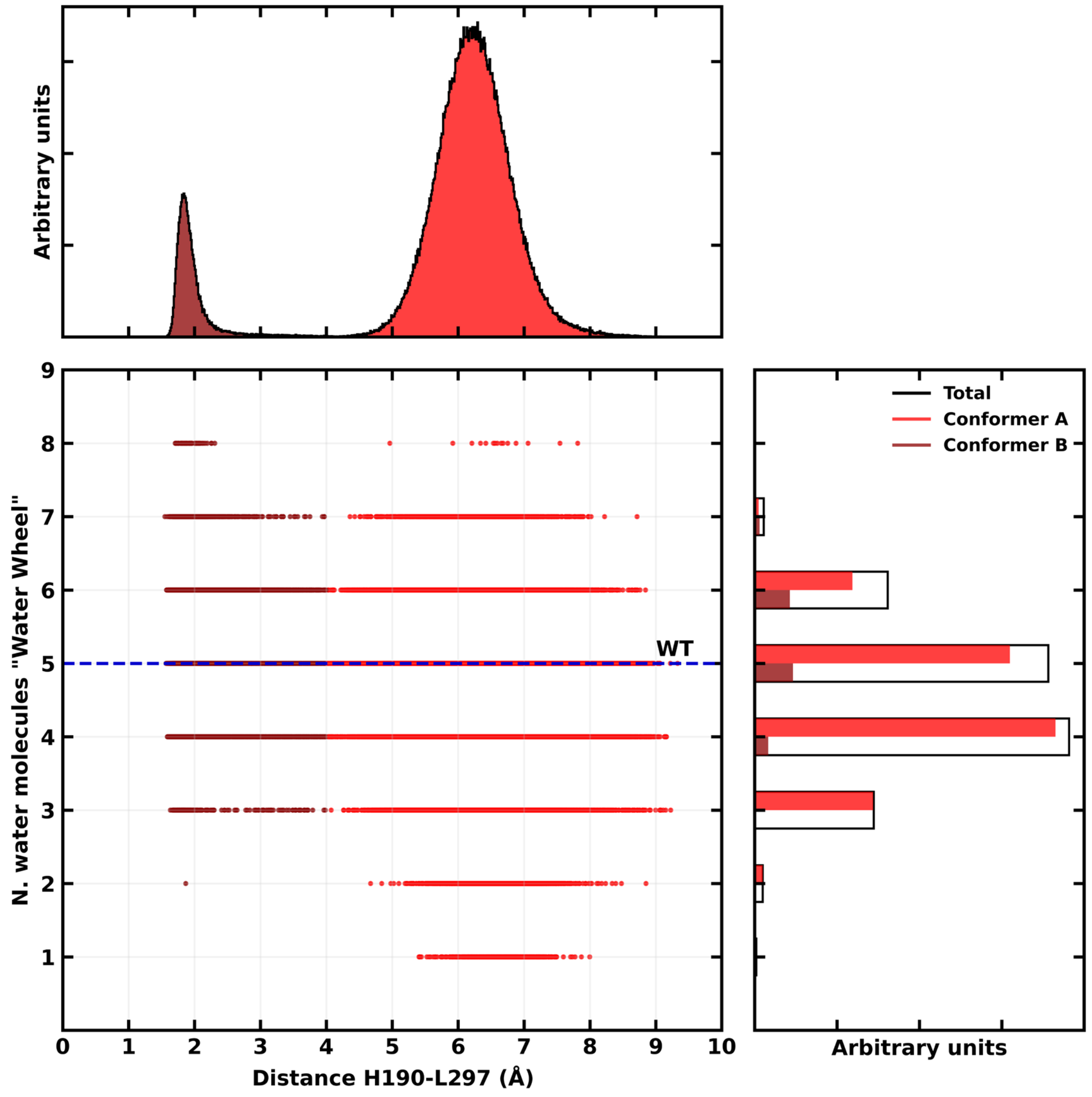

Supplementary Fig. 26 H190-L297 conformers vs. water wheel occupancy. The scatter plot in the central panel shows the number of sampled water molecules in the "water wheel" region as a function of the H190-L297 distance. Data points are colored according to the conformer. The blue dashed line indicates the WT average. Top and right panels show the marginal distributions for distance and water occupancy, respectively, with total (black), Conformer A (red), and Conformer B (dark red) contributions. The plot shows how the two conformers of H190-L297 can affect the number of water molecules for the water wheel as defined in Fig. 7 (panel c) of the article. In Conformer A (no H-bond), the main population shifts to lower water counts, suggesting either water molecules leave the 'wheel' to form a hydrogen bond with H190, or that the entire cluster translates accordingly, in line with the distance distribution shown in Supplementary Fig. 22.

5. References

1. Debus, R.J. Evidence from FTIR Difference Spectroscopy That D1-Asp61 Influences the Water Reactions of the Oxygen-Evolving Mn_4CaO_5 Cluster of Photosystem II. *Biochemistry* **53**, 2941-2955 (2014).
2. Berthomieu, C., Nabedryk, E., Mantele, W. & Breton, J. Characterization by FTIR spectroscopy of the photoreduction of the primary quinone acceptor Q_A in photosystem II. *FEBS Lett.* **269**, 363-367 (1990).
3. Gisriel, C.J. et al. High-resolution cryo-electron microscopy structure of photosystem II from the mesophilic cyanobacterium, *Synechocystis* sp. PCC 6803. *Proc. Natl. Acad. Sci. U.S.A.* **119**, e2116765118 (2022).
4. Bhowmick, A. et al. Structural evidence for intermediates during O_2 formation in photosystem II. *Nature* **617**, 629–636 (2023).
5. Maier, J.A. et al. ff14SB: improving the accuracy of protein side chain and backbone parameters from ff99SB. *J Chem Theory Comput* **11**, 3696-3713 (2015).
6. Narzi, D., Coccia, E., Manzoli, M. & Guidoni, L. Impact of molecular flexibility on the site energy shift of chlorophylls in Photosystem II. *Biophys. Chem.* **229**, 93-98 (2017).
7. Bovi, D., Narzi, D. & Guidoni, L. Magnetic interactions in the catalyst used by nature to split water: a DFT + *U* multiscale study on the Mn_4CaO_5 core in photosystem II. *New J Phys* **16**, 015020 (2014).
8. Wang, J.M., Wolf, R.M., Caldwell, J.W., Kollman, P.A. & Case, D.A. Development and testing of a general amber force field. *J. Comput. Chem.* **25**, 1157-1174 (2004).
9. Frisch, M.J. Gaussian 16 Revision C.01. *Gaussian Inc. Wallingford CT* (2016).
10. Narzi, D., Capone, M., Bovi, D. & Guidoni, L. Evolution from S_3 to S_4 state of the oxygen evolving complex in Photosystem II monitored by QM/MM dynamics. *Chem. Eur. J.* (2018).
11. Cárdenas, G., Marquetand, P., Mai, S. & González, L. A force field for a manganese-vanadium water oxidation catalyst: redox potentials in solution as showcase. *Catalysts* **11**, 493 (2021).
12. Neese, F. The ORCA program system. *Wiley Interdisciplinary Reviews: Computational Molecular Science* **2**, 73-78 (2012).
13. Van der Spoel, D. et al. GROMACS: Fast, flexible, and free. *J. Comput. Chem.* **26**, 1701-1718 (2005).
14. Jämbeck, J.P. & Lyubartsev, A.P. Derivation and systematic validation of a refined all-atom force field for phosphatidylcholine lipids. *J. Phys. Chem. B* **116**, 3164-3179 (2012).
15. Jorgensen, W.L., Chandrasekhar, J., Madura, J.D., Impey, R.W. & Klein, M.L. Comparison of Simple Potential Functions for Simulating Liquid Water. *J. Chem. Phys.* **79**, 926-935 (1983).
16. Berendsen, H.J., Postma, J.v., Van Gunsteren, W.F., DiNola, A. & Haak, J.R. Molecular dynamics with coupling to an external bath. *J. Chem. Phys.* **81**, 3684-3690 (1984).
17. Darden, T., York, D. & Pedersen, L. Particle mesh Ewald: An N*log(N) method for Ewald sums in large systems. *J. Chem. Phys.* **98**, 10089-10092 (1993).
18. Hess, B., Bekker, H., Berendsen, H.J.C. & Fraaije, J.G.E.M. LINCS: A linear constraint solver for molecular simulations. *J. Comput. Chem.* **18**, 1463-1472 (1997).
19. Bussi, G., Donadio, D. & Parrinello, M. Canonical sampling through velocity rescaling. *J. Chem. Phys.* **126** (2007).
20. Nose, S. & Klein, M.L. Constant Pressure Molecular-Dynamics for Molecular-Systems. *Mol Phys* **50**, 1055-1076 (1983).
21. Parrinello, M. & Rahman, A. Polymorphic Transitions in Single-Crystals: A New Molecular Dynamics Method. *Journal of Applied Physics* **52**, 7182-7190 (1981).
22. Laurent, B. et al. Epock: rapid analysis of protein pocket dynamics. *Bioinformatics* **31**, 1478-1480 (2015).
23. Humphrey, W., Dalke, A. & Schulten, K. VMD: visual molecular dynamics. *J Mol Graph* **14**, 33-38, 27-38 (1996).
24. Hussein, R. et al. Cryo–electron microscopy reveals hydrogen positions and water networks in photosystem II. *Science* **384**, 1349-1355 (2024).
