# Supplementary material for: Three rate-determining protein roles in photosynthetic O2-evolution addressed by time-resolved experiments on genetically modified photosystems
Source: Nat Commun. 2025 Oct 28;16:9515. doi: 10.1038/s41467-025-64513-9 (PMC12569156; doi:10.1038/s41467-025-64513-9)
Supplement: Supplementary file 2 — Description of Additional Supplementary Files [file 41467_2025_64513_MOESM2_ESM.pdf]

Description of Additional Supplementary Files

File Name: Supplementary Movie 1

Description: The movie shows rapid water movements around the Mn_4Ca-oxo complex and its ligand environment, including the water-wheel region, in the wild-type system. The visualization illustrates the high mobility of water on the picosecond timescale.

Technical details: The movie was prepared using a representative 10 ns segment (460–470 ns) of the MD trajectory from the wild-type system (monomer 1), sampled over 1000 frames. Trajectory smoothing (window size = 10) was applied to improve visual continuity. Frames were rendered in VMD5 and then compiled into a movie for visualization purposes.
