# Supplementary material for: Three rate-determining protein roles in photosynthetic O2-evolution addressed by time-resolved experiments on genetically modified photosystems
Source: Nat Commun. 2025 Oct 28;16:9515. doi: 10.1038/s41467-025-64513-9 (PMC12569156; doi:10.1038/s41467-025-64513-9)
Supplement: Supplementary file 4 — Reporting Summary [file 41467_2025_64513_MOESM4_ESM.pdf]

nature portfolio

Corresponding author(s): Holger Dau

Last updated by author(s): Sep 11, 2025

Reporting Summary

Nature Portfolio wishes to improve the reproducibility of the work that we publish. This form provides structure for consistency and transparency in reporting. For further information on Nature Portfolio policies, see our Editorial Policies and the Editorial Policy Checklist.

Statistics

For all statistical analyses, confirm that the following items are present in the figure legend, table legend, main text, or Methods section.

n/a	Confirmed	
☐	☒	The exact sample size (*n*) for each experimental group/condition, given as a discrete number and unit of measurement
☐	☒	A statement on whether measurements were taken from distinct samples or whether the same sample was measured repeatedly
☒	☐	The statistical test(s) used AND whether they are one- or two-sided<br>*Only common tests should be described solely by name; describe more complex techniques in the Methods section.*
☒	☐	A description of all covariates tested
☒	☐	A description of any assumptions or corrections, such as tests of normality and adjustment for multiple comparisons
☐	☒	A full description of the statistical parameters including central tendency (e.g. means) or other basic estimates (e.g. regression coefficient) AND variation (e.g. standard deviation) or associated estimates of uncertainty (e.g. confidence intervals)
☒	☐	For null hypothesis testing, the test statistic (e.g. *F*, *t*, *r*) with confidence intervals, effect sizes, degrees of freedom and *P* value noted<br>*Give P values as exact values whenever suitable.*
☒	☐	For Bayesian analysis, information on the choice of priors and Markov chain Monte Carlo settings
☒	☐	For hierarchical and complex designs, identification of the appropriate level for tests and full reporting of outcomes
☒	☐	Estimates of effect sizes (e.g. Cohen's *d*, Pearson's *r*), indicating how they were calculated

Our web collection on statistics for biologists contains articles on many of the points above.

Software and code

Policy information about availability of computer code

Data collection	Only instrument-specific code was used that is irrelevant for our results and conclusions.
Data analysis	Python code (Python 3.7.16) was used to correct the time-resolved IR data for S-state starting populations and cycle inefficiency (miss factor) and to fit the data globally to a sum of exponentials. This code is analogous to code deposited previously (doi: 10.5281/zenodo.7682034). Standard GROMACS packages were used for the MD simulations, which are generally available - see also Van der Spoel et al., 2005 (ref. 83).

For manuscripts utilizing custom algorithms or software that are central to the research but not yet described in published literature, software must be made available to editors and reviewers. We strongly encourage code deposition in a community repository (e.g. GitHub). See the Nature Portfolio guidelines for submitting code & software for further information.

Data

Policy information about availability of data

All manuscripts must include a data availability statement. This statement should provide the following information, where applicable:

- Accession codes, unique identifiers, or web links for publicly available datasets
- A description of any restrictions on data availability
- For clinical datasets or third party data, please ensure that the statement adheres to our policy

The time-resolved IR data obtained for 10 consecutive excitation flashes (applied to wild-type PSII, N298A and D61A) is availably at Zenodo (https://doi.org/10.5281/zenodo.15789665). The mass spectrometry data are available via ProteomeXchange with identifier PXD068038. Source data for figures 2, 3, 4, 5, 7b,c and 8b,d,g,e are provided with the paper.

Research involving human participants, their data, or biological material

Policy information about studies with human participants or human data. See also policy information about sex, gender (identity/presentation), and sexual orientation and race, ethnicity and racism.

Reporting on sex and gender	N/A
Reporting on race, ethnicity, or other socially relevant groupings	N/A
Population characteristics	N/A
Recruitment	N/A
Ethics oversight	N/A

Note that full information on the approval of the study protocol must also be provided in the manuscript.

Field-specific reporting

Please select the one below that is the best fit for your research. If you are not sure, read the appropriate sections before making your selection.

- [x] Life sciences
- [ ] Behavioural & social sciences
- [ ] Ecological, evolutionary & environmental sciences

For a reference copy of the document with all sections, see nature.com/documents/nr-reporting-summary-flat.pdf

Life sciences study design

All studies must disclose on these points even when the disclosure is negative.

Sample size	IR data: For each IR data set between 500-5000 flash cycles were acquired and averaged; these flash cycles were measured on 1-7 distinct samples per data set.<br>O2 polarography: Per temperature and PSII variant, at least three distinct data sets were acquired, each of which is comprised of 70-80 flash-induced O2 transients. All 210-240 O2 transients were averaged for better signal-to-noise prior to fitting.
Data exclusions	IR data: Individual flash transients were excluded if the detected signal was below or above a certain threshold. Transients with poor S/N due to sinusoidal noise (e.g. 50 Hz) were also excluded.
Replication	IR data: For each PSII variant (wild-type, N298A, D61A) three repeats (n=3) were measured at least at one wavenumber, demonstrating that the data was in principle reproducible. Due to a limited amount of sample, it was not possible to measure on distinct samples at all wavenumbers.<br>O2 polarography: At least three replicates per data set were acquired; visual inspection showed that the data sets agreed with each other, thus ensuring reproducibility. The three replicates were averaged prior to fitting for better signal-to-noise.
Randomization	N/A: The topic of our study is a biological one, but the experimental biophysical approach and methodology is typical for physical sciences. It is not an empirical investigation where randomization can be applied.
Blinding	N/A: The topic of our study is a biological one, but the experimental biophysical approach and methodology is typical for physical sciences. It is not an empirical investigation where blinding can be applied.

Reporting for specific materials, systems and methods

We require information from authors about some types of materials, experimental systems and methods used in many studies. Here, indicate whether each material, system or method listed is relevant to your study. If you are not sure if a list item applies to your research, read the appropriate section before selecting a response.

Materials & experimental systems

n/a	Involved in the study
☒	☐ Antibodies
☒	☐ Eukaryotic cell lines
☒	☐ Palaeontology and archaeology
☒	☐ Animals and other organisms
☒	☐ Clinical data
☒	☐ Dual use research of concern
☒	☐ Plants

Methods

n/a	Involved in the study
☒	☐ ChIP-seq
☒	☐ Flow cytometry
☒	☐ MRI-based neuroimaging

Plants

Seed stocks	N/A
Novel plant genotypes	N/A
Authentication	N/A
